# A Stochastic Version of the Jansen and Rit Neural Mass Model: Analysis and Numerics

**DOI:** 10.1186/s13408-017-0046-4

**Published:** 2017-08-08

**Authors:** Markus Ableidinger, Evelyn Buckwar, Harald Hinterleitner

**Affiliations:** Johannes Kepler University Linz, Altenberger Straße 69, Linz, 4040 Austria

**Keywords:** Jansen and Rit neural mass model, Stochastic Hamiltonian system, Asymptotic behaviour, Stochastic splitting schemes

## Abstract

Neural mass models provide a useful framework for modelling mesoscopic neural dynamics and in this article we consider the Jansen and Rit neural mass model (JR-NMM). We formulate a stochastic version of it which arises by incorporating random input and has the structure of a damped stochastic Hamiltonian system with nonlinear displacement. We then investigate path properties and moment bounds of the model. Moreover, we study the asymptotic behaviour of the model and provide long-time stability results by establishing the geometric ergodicity of the system, which means that the system—independently of the initial values—always converges to an invariant measure. In the last part, we simulate the stochastic JR-NMM by an efficient numerical scheme based on a splitting approach which preserves the qualitative behaviour of the solution.

## Introduction

Neural mass models have been studied as models describing coarse grained activity of large populations of neurons [1–7] since the 1970s. They have successfully been used to fit neuroimaging data, understanding EEG rhythms [8] or epileptic brain dynamics [9], and are now also a major building block in the Virtual Brain [10]. For a summary on their history, applications and an outlook on their future possible use, we refer to [11]. In general, neural mass models can be derived as a mean-field limit from microscopic models [12] and involve just a few state variables such as average membrane potentials and average population firing rates.

In this article, we focus on the Jansen and Rit neural mass model (JR-NMM) [13], which has been introduced as a model in the context of human electroencephalography (EEG) rhythms and visual evoked potentials [14]. It dates back to the work of Lopes da Silva and Van Rotterdam [3, 5, 15]. The JR-NMM is a biologically motivated convolution-based model of a neuronal population involving two subpopulations, i.e. excitatory and inhibitory interneurons forming feedback loops, which can describe background activity, alpha activity, sporadic and also rhythmic epileptic activity.

The original JR-NMM is formulated as a set of three coupled second-order nonlinear ordinary differential equations (ODEs), i.e. these constitute a system of coupled nonlinear oscillators, often rewritten as the six-dimensional system of first-order equations. After introducing this system in Sect. 2, we rewrite the system in the format of classical mechanics, that is, as a damped Hamiltonian system with a nonlinear displacement. Furthermore, in most of the literature, the JR-NMM includes a term representing extrinsic input or background noise, which essentially is done by declaring that input function to be a stochastic process. Mathematically this implies that the solution process of the ODE system then also is a stochastic process inheriting the analytical properties of the input process and requiring some framework of stochastic analysis for its mathematical treatment. In Sect. 3 we discuss options for such a framework and in this article we choose to formulate a stochastic JR-NMM as a stochastic differential equation (SDE) with additive noise, in particular a stochastic damped Hamiltonian system with a nonlinear term. Systems of SDEs of this or similar form are well studied in the molecular dynamics literature, where they are often called Langevin equations.^1^ In this article we provide a range of results employing various techniques available in the framework of stochastic analysis developed for SDEs: In Sect. 4 we establish basic properties of the SDE such as moment bounds and bounds on the path behaviour. Section 5 augments existing analysis of the dynamics of the deterministic JR-NMM, in particular we consider stochastic versions of equilibrium solutions, i.e. invariant measures, as well as the long-time behaviour of solutions of the SDE with respect to this invariant measure. These results may be interpreted as starting points for studies of phenomenological stochastic bifurcations or noise-induced transitions. Finally, in Sect. 6, we present efficient numerical methods designed for stochastic Hamiltonian problems and show that these numerical methods, which represent discrete stochastic systems for any fixed step-size, respect the properties previously established for the SDE system (subject to mild conditions on the step-size). Thus the resulting numerical methods will not only be quite efficient for future computational studies with the stochastic JR-NMM, they also provide *reliable* computational results.

## Description of the Original Jansen and Rit Neural Mass Model

A detailed summary of the model derivation from the neuroscientific point of view can be found in [18–20]. The main neural population, the excitatory and inhibitory interneurons, are in each case described by both a second-order ordinary differential operator, which transforms the mean incoming firing rate into the mean membrane potential, and a nonlinear function, which transforms the mean membrane potential into the mean output firing rate. For $t\in[0,T]$ with $T\in\mathbb{R}^{+}$, the JR-NMM proposed in [13] consists of three coupled nonlinear ODEs of second order

1 $$\begin{aligned} \ddot{x}_{0}(t)&=Aa\operatorname{Sigm} \bigl(x_{1}(t)-x_{2}(t)\bigr)-2a\dot{x}_{0}(t)-a^{2} x_{0}(t) , \\ \ddot{x}_{1}(t)&=Aa\bigl[p(t)+C_{2}\operatorname{Sigm} \bigl(C_{1}x_{0}(t)\bigr)\bigr]-2a\dot {x}_{1}(t)-a^{2}x_{1}(t), \\ \ddot{x}_{2}(t)&=BbC_{4}\operatorname{Sigm} \bigl(C_{3}x_{0}(t)\bigr)-2b\dot {x}_{2}(t)-b^{2}x_{2}(t), \end{aligned}$$

which can be written as the six-dimensional first-order ODE system

2 $$ \begin{aligned}\dot{x}_{0}(t)&=x_{3}(t), \\ \dot{x}_{1}(t)&=x_{4}(t), \\ \dot{x}_{2}(t)&=x_{5}(t), \\ \dot{x}_{3}(t)&=Aa\operatorname{Sigm}\bigl(x_{1}(t)-x_{2}(t) \bigr)-2ax_{3}(t)-a^{2} x_{0}(t), \\ \dot{x}_{4}(t)&=Aa\bigl[p(t)+C_{2}\operatorname {Sigm} \bigl(C_{1}x_{0}(t)\bigr)\bigr]-2ax_{4}(t)-a^{2}x_{1}(t) , \\ \dot{x}_{5}(t)&=BbC_{4}\operatorname {Sigm} \bigl(C_{3}x_{0}(t)\bigr)-2bx_{5}(t)-b^{2}x_{2}(t), \end{aligned}$$

with initial value $(x_{0}(0),\dots,x_{5}(0))^{T}=\mathbf{x_{0}}\in \mathbb{R}^{6}$. Here, $x_{i}$ for $i\in\{0,1,2\}$ describe the mean postsynaptic potentials of distinct neuronal populations. The *output signal* $y(t):=x_{1}(t)-x_{2}(t)$ describes the average membrane potential of the main family, i.e. the principal neurons of the JR-NMM (see [18, 19, 21]). The function $p:[0,T]\to\mathbb{R}$ describes the external input which may originate both from external sources or the activity of neighbouring neural populations. We will discuss the mathematical modelling of *p* in more detail at the end of this section. The sigmoid function $\operatorname {Sigm}:\mathbb{R}\to[0,\nu_{\max}],\nu_{\max}>0$ (as suggested in [4]) is given by

$$\begin{aligned} \operatorname {Sigm}(x):=\frac{\nu_{\max}}{1+\mathrm {e}^{r(v_{0}-x)}} \end{aligned}$$

and works as a gain function transforming the average membrane potential of a neural population into an average firing rate (see [22, 23]). The constant $\nu_{\max}$ denotes the maximum firing rate of the neural population, $v_{0}\in \mathbb {R}$ is the value for which 50% of the maximum firing rate is attained and $r>0$ determines the slope of the sigmoid function at $v_{0}$.

System (2) includes 11 parameters *A*, *B*, *a*, *b*, $C_{1}$, $C_{2}$, $C_{3}$, $C_{4}$, $\nu_{\max}$, *r*, $v_{0}$ and typical values for these parameters, taken from [13, 19], are given in Table 1. The parameters *A*, *B*, *a* and *b* model basic features of postsynaptic potentials. In particular, *A* and *B* denote the excitatory and inhibitory synaptic gain, respectively, and $a^{-1}$ and $b^{-1}$ are corresponding time constants. The connectivity constants $C_{i}$ for $i\in \lbrace1,2,3,4\rbrace$, modelling the interactions between the main population and interneurons, are assumed to be proportional to a single parameter *C* which characterises the average number of synapses between populations (see [13]). The solution behaviour of System (2) depends sensitively on the values of the parameters (we refer to the bifurcation analyses in [18, 19, 24]). Especially, changes in the connectivity constants $C_{i}$ can result in drastic changes of the solution path.

**Table 1:** **Typical values established in the original JR-NMM [** 13 **] taken from [** 19 **]**

Parameter	Description	Typical value
*A*	Average excitatory synaptic gain	3.25 mV
*B*	Average inhibitory synaptic gain	22 mV
$a^{-1}$	Time constant of excitatory postsynaptic potential	10 ms
$b^{-1}$	Time constant of inhibitory postsynaptic potential	20 ms
*C*	Average number of synapses between the populations	135
$C_{1}$	Avg. no. of syn. established by principal neurons on excitatory interneurons	C
$C_{2}$	Avg. no. of syn. established by excitatory interneurons on principal neurons	0.8 C
$C_{3}$	Avg. no. of syn. established by principal neurons on inhibitory interneurons	0.25 C
$C_{4}$	Avg. no. of syn. established by inhibitory interneurons on principal neurons	0.25 C
$\nu_{\max}$	Maximum firing rate of the neural populations (max. of sigmoid fct.)	5 s^−1^
$v_{0}$	Value for which 50% of the maximum firing rate is attained	6 mV
*r*	Slope of the sigmoid function at $v_{0}$	0.56 mV^−1^

Subsequently, we will employ the Hamiltonian formulation of classical mechanics to study coupled oscillators such as System (1) or (2). Let $Q:=(x_{0},x_{1},x_{2})^{T}$ and $P:=(x_{3},x_{4},x_{5})^{T}$ denote three-dimensional vectors, then System (2) can be written as a *damped Hamiltonian system with nonlinear displacement*,

3 $$ \begin{aligned} \frac{dQ}{dt}&=\nabla_{P} H(Q,P), \\ \frac{dP}{dt}&=-\nabla_{Q}H(Q,P)- 2\varGamma P+ G(t,Q) . \end{aligned}$$

In this formulation, the system consists of a *Hamiltonian part* with Hamiltonian function $H:\mathbb{R}^{6}\to\mathbb{R}^{+}_{0}$,

$$\begin{aligned} H(Q,P):=\frac{1}{2} \bigl(\Vert P\Vert _{\mathbb{R}^{3}}^{2}+ \Vert \varGamma Q\Vert _{\mathbb {R}^{3}}^{2} \bigr), \end{aligned}$$

a *damping part* with damping matrix $\varGamma=\operatorname {diag}[a,a,b]\in\mathbb{R}^{3\times3}$, and a *nonlinear part* given by the function $G:[0,T]\times\mathbb{R}^{3}\to\mathbb{R}^{3}$, with

$$\begin{aligned} G(t,Q):= \bigl( Aa \operatorname {Sigm}(x_{1}-x_{2}), Aa \bigl[p(t)+C_{2} \operatorname {Sigm}(C_{1} x_{0})\bigr], BbC_{4} \operatorname {Sigm}(C_{3}x_{0}) \bigr)^{T}. \end{aligned}$$

The Hamiltonian $H(Q,P)$ may be interpreted as the total energy of an electrical RCL parallel resonant circuit; see [22]. In particular, $H(Q,P)$ is proportional to the sum of the inductive and capacitive energy of the neuronal population, respectively. If the input $p(t)$ is a bounded deterministic function, the solution curve and the total energy $H(Q,P)$ are bounded (this is an immediate result of Theorem 4.2 in Sect. 4) and the time change in the total energy is given by

$$\begin{aligned} \frac{d}{dt}H(Q,P)=-2 P^{T} \varGamma P+ P^{T}G(t,Q) . \end{aligned}$$

In the original paper by Jansen and Rit [13], the external input $p(t)$ has been used to represent spontaneous background noise as well as peak-like functions for generating evoked potentials. In the latter case the extrinsic input has been modelled as a deterministic periodic function (see also [25]) and with this type of input, the solution of the System (1) (or (2) or (3)) remains a deterministic function and the mathematical background to treat it is deterministic analysis. In the former case, i.e. when $p(t)$ represents spontaneous background noise and is modelled as a stochastic process, the mathematical background immediately changes to be stochastic analysis. In particular, the solution of the Systems (1), (2) or (3) becomes a stochastic process and it inherits the mathematical properties of the input process $p(t)$. Within stochastic analysis, (1), (2) or (3) may be interpreted in different frameworks, with consequences depending on the specific choices of $p(t)$.

(i) Random Ordinary Differential Equation (RODE) framework: RODEs are pathwise ODEs involving a stochastic process in their right-hand side, i.e. for a sufficiently smooth function $f:\mathbb {R}^{m}\times\mathbb{R}^{d} \rightarrow\mathbb{R}^{d}$ and an *m*-dimensional stochastic process $\xi(t)$, a *d*-dimensional system of RODEs is given by
  $$\dot{x}(t)= f\bigl(\xi(t),x(t)\bigr) , $$
  with an appropriate initial value. One may then choose the stochastic input process for example as a Wiener process or a coloured noise process, these processes exist in the classical sense and have almost surely continuous paths. In this framework standard deterministic analysis for e.g. guaranteeing existence and uniqueness of solutions can be applied pathwise; see for example [26], Chap. 1. However, the solution of this equation inherits the smoothness properties of the driving stochastic process $\xi(t)$, independent of the smoothness of the function *f*. Analysis of properties and dynamics of solutions of RODEs may be performed pathwise by standard analysis techniques, bearing in mind that the low smoothness of the solutions limits the applicability of many classical results, such as Taylor’s theorem. We further refer to [27] for relevant results concerning random dynamical systems. Another consequence concerns the numerical treatment: as the order of convergence of classical numerical schemes for ODEs is determined by the smoothness of the solution of that ODE, when such schemes are applied pathwise to RODEs, they usually converge with a lower order than their expected one. In particular, they converge with order at most $1/2$ when the input process is chosen as the Wiener process or a coloured noise process, as their paths are only Hölder continuous of order less than $1/2$. We refer to [28] and its references for further information on numerical methods specifically designed for RODEs.
(ii) Stochastic Differential Equation framework: If one were to choose the stochastic input process in an RODE as above as a Gaussian white noise process, one would need to deal with the fact that such a process exists only in the sense of *generalised stochastic processes*; see [29], Sect. 3.2, or [30], Appendix I. In particular, Gaussian white noise is usually interpreted as the (generalised) derivative of the Wiener process, which itself is almost surely nowhere differentiable in the classical sense. It is much more convenient to work in the classical stochastic analysis framework designed to deal with differential equations ‘subject to (white) noise’ and interpret Systems (1), (2) or (3) as a stochastic differential equation; see also [29], Sect. 4.1. A considerable amount of results concerning analysis, dynamics, numerics, statistics, etc. of SDEs is available and for stochastic numerics we refer for example to [31], which also treats SDEs driven by coloured noise.

## Jansen and Rit Neural Mass Model as a Damped Stochastic Hamiltonian System with Nonlinear Displacement

Let $(\varOmega, \mathcal {F},\mathbb {P})$ be a complete probability space together with the filtration $\{\mathcal {F}_{t}\}_{t\in[0,T]}$ which is right-continuous and complete. We extend the model of System (2) by allowing perturbation terms such as $p(t)$ not only in $x_{1}(t)$ but in both $x_{0}(t)$ and $x_{2}(t)$ as well. For this purpose, we define the functions $\mu_{i}:[0,T]\to\mathbb{R}$ and $\sigma_{i}:[0,T]\to \mathbb{R}^{+}$ for $i\in\lbrace3,4,5\rbrace$. The functions $\mu_{i}$ will be used for representing deterministic input whereas $\sigma_{i}$ will be used for scaling the stochastic components. In an analogous way to the exposition concerning stochastic oscillators in [32], Chap. 8, or in [33], Chap. 14.2, we symbolically introduce Gaussian white noise $\dot{W}_{i}$ representing the stochastic input into Eq. (1) as follows:

4 $$ \begin{aligned} dX_{0}(t)={}&X_{3}(t)\,dt, \\ dX_{1}(t)={}&X_{4}(t)\,dt, \\ dX_{2}(t)={}&X_{5}(t)\,dt, \\ dX_{3}(t)={}&\bigl[Aa\bigl[\mu_{3}(t)+\operatorname {Sigm} \bigl(X_{1}(t)-X_{2}(t)\bigr)\bigr]-2aX_{3}(t)-a^{2} X_{0}(t)\bigr]\,dt \\ &{}+\sigma_{3}(t)\,dW_{3}(t), \\ dX_{4}(t)={}&\bigl[Aa\bigl[\mu_{4}(t)+C_{2} \operatorname {Sigm}\bigl(C_{1}X_{0}(t)\bigr) \bigr]-2aX_{4}(t)-a^{2}X_{1}(t)\bigr]\,dt \\ &{}+ \sigma_{4}(t)\,dW_{4}(t), \\ dX_{5}(t)={}&\bigl[Bb\bigl[\mu_{5}(t)+C_{4} \operatorname {Sigm}\bigl(C_{3}X_{0}(t)\bigr) \bigr]-2bX_{5}(t)-b^{2}X_{2}(t)\bigr]\,dt\\ &{}+ \sigma_{5}(t)\,dW_{5}(t), \end{aligned}$$

with deterministic initial value $(X_{0}(0),\dots,X_{5}(0))^{T}=\mathbf {X_{0}}\in\mathbb{R}^{6}$. Here, the processes $W_{i}(t)$ for $i\in\{ 3,4,5\}$ are independent, $\mathcal{F}_{t}$-adapted Wiener processes on $(\varOmega,\mathcal{F},\mathbb{P})$. Note that as the system above is an additive noise system the Itô and Stratonovich interpretations of that SDE system coincide. As for the deterministic case in Sect. 2, we can use the $(Q,P)$-notation of classical mechanics

5 $$ \begin{aligned}dQ(t)&=\nabla_{P} H(Q,P)\,dt, \\ dP(t)&= \bigl(-\nabla_{Q}H(Q,P)- 2\varGamma P+ G(t,Q) \bigr)\,dt+ \varSigma(t)\,dW(t) , \end{aligned}$$

with initial values

$$\begin{aligned} Q(0)&=\bigl(X_{0}(0),X_{1}(0),X_{2}(0) \bigr)^{T}=Q_{0}\in\mathbb{R}^{3}\quad \mbox{and} \\ P(0)&=\bigl(X_{3}(0),X_{4}(0),X_{5}(0) \bigr)^{T}=P_{0}\in\mathbb{R}^{3}, \end{aligned}$$

diffusion matrix

$$\varSigma(t)=\operatorname{diag}\bigl[\sigma_{3}(t), \sigma_{4}(t),\sigma _{5}(t)\bigr]\in\mathbb{R}^{3\times3}, $$

and nonlinear displacement

$$G(t,Q):= \begin{pmatrix} Aa[\mu_{3}(t)+\operatorname {Sigm}(X_{1}-X_{2})]\\ Aa[\mu_{4}(t)+C_{2}\operatorname {Sigm}(C_{1} X_{0})]\\ Bb[\mu_{5}(t)+C_{4}\operatorname {Sigm}(C_{3}X_{0})] \end{pmatrix} . $$

As before, we define the output signal as $Y(t)=X_{1}(t)-X_{2}(t)$.

Systems of the type (5), typically called Langevin equations, have received considerable attention in the literature of molecular dynamics (see [16] for an overview). In particular, the long-time properties of such systems have been intensively studied in [34–36]. We employ these techniques in Sect. 5 to study the long-time behaviour of System (5).

We briefly discuss the existence of a solution of Eq. (5). As the sigmoid function Sigm is globally Lipschitz continuous, the existence and pathwise uniqueness of an $\mathcal {F}_{t}$-adapted solution is a standard result; see e.g. in [29], Theorem 6.2.2. In particular, *Q* is continuously differentiable. In the current context, it makes sense to assume that the functions $\mu_{i}$ and $\sigma_{i}$ are smooth and bounded which we will do in the following.

**Fig. 1:**
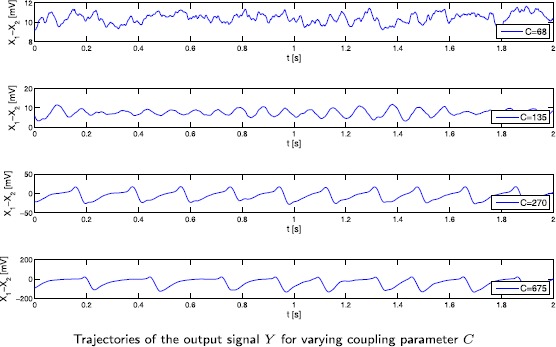
Output signal *Y*

**Fig. 2:**
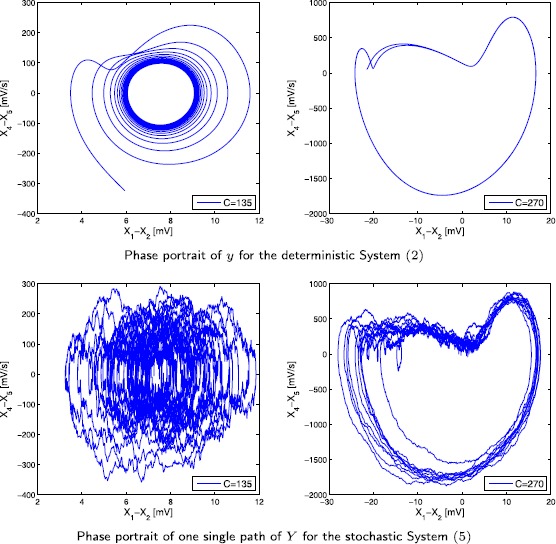
Phase portraits

We simulate the solution of Eq. (5) with the splitting integrator (24) proposed in Sect. 6 and illustrate the output signal in Fig. 1. The coefficients and the noise components are chosen in such a way that the simulation results of [14] for varying connectivity constants *C* can be reproduced. The numerical values for the parameters are given in Table 1. For the deterministic part of the external inputs we set $\mu_{3}=\mu_{5}=0$ and $\mu_{4}=220$, for the diffusion components we set $\sigma_{3}=\sigma _{5}=10$ and $\sigma_{4}=1{,}000$ such that ‘weak noise’ is acting on the components $X_{3}$ and $X_{5}$; $X_{4}$ receives a stronger noise input. As in the original paper [14] we see (noisy) *α*-rhythm-like behaviour as well as spiking behaviour for varying connectivity constants *C*. In Fig. 2 we provide an illustration of changes in the system behaviour induced by including noise with plots of the phase portrait of the output signal for the case $C=135$ and $C=270$. The top two pictures show simulations of *y* of System (2), i.e. without noise, where the solution curves quickly converge towards a limit cycle. The bottom two pictures show a path of *Y* of System (5) and in particular for $C=135$, the behaviour of the path is markedly different from the deterministic case.

## Moment Bounds and Path Behaviour

We have already mentioned in Sect. 2 that the solution paths of Eq. (1) take values in a bounded set. It is natural to ask in which sense this behaviour transfers to the stochastic setting. We answer this question via a twofold strategy. On the one hand we will study the time evolution of the moments of the solution, which describes the average behaviour of all solution paths. On the other hand we will study the behaviour on the level of single paths and estimate the probability that a specific path exceeds a given threshold. Before we study these qualitative properties of Eq. (5) we provide a convolution-based representation for the *Q*-component of Eq. (5) which simplifies the corresponding calculations considerably.

### Convolution-Based Representation of the JR-NMM

In this section we rewrite Eq. (5) using $X=(Q,P)^{T}$ as

6 $$ dX(t)=\bigl(MX(t)+N\bigl(t,X(t)\bigr)\bigr)\,dt+S(t)\,dW(t), $$

where

$$\begin{aligned} M =& \begin{pmatrix} \mathbb{O}_{3} & \mathbb{I}_{3}\\ -\varGamma^{2} & -2 \varGamma \end{pmatrix} , \\ N\bigl(t,X(t)\bigr) =& \begin{pmatrix} 0_{3}\\ G(t,Q(t)) \end{pmatrix} \quad \text{and} \\ S(t) =& \begin{pmatrix} \mathbb{O}_{3}\\ \varSigma(t) \end{pmatrix} . \end{aligned}$$

Here, we denote by $\mathbb{O}_{3},\mathbb{I}_{3} \in\mathbb {R}^{3\times3}$ the zero and identity matrix, respectively. Moreover, we define $0_{3}:=(0,0,0)^{T}$ and $1_{3}:=(1,1,1)^{T}$.

Note that *M* is a block matrix with diagonal submatrices. Hence, we can calculate an explicit expression for the matrix exponential,

7 $$\begin{aligned} \mathrm {e}^{Mt} =& \begin{pmatrix} \mathrm {e}^{-\varGamma t}(\mathbb{I}_{3}+\varGamma t) & \mathrm {e}^{-\varGamma t}t\\ -\varGamma^{2}\mathrm {e}^{-\varGamma t}t & \mathrm {e}^{-\varGamma t}(\mathbb{I}_{3}-\varGamma t) \end{pmatrix} \\ =:& \begin{pmatrix} \vartheta(t) & \kappa(t) \\ \vartheta'(t) & \kappa'(t) \end{pmatrix} . \end{aligned}$$

Obviously, the matrix exponential fulfils $e^{Mt}M=Me^{Mt}$. This allows one to represent solutions of Eq. (5) via the following convolution-based formula.

#### Theorem 4.1

*The component* *Q* *of the unique solution of Eq*. (5) *solves for* $t\in[0,T]$ *the integral equation*

8 $$\begin{aligned} Q(t) =&\vartheta(t)Q_{0}+\kappa(t)P_{0}+ \int_{0}^{t} \kappa(t-s) G\bigl(s,Q(s)\bigr)\,ds \\ &{}+ \int _{0}^{t} \kappa(t-s) \varSigma(s)\,dW(s). \end{aligned}$$

*We call Eq*. (8) *the convolution*-*based representation of* *Q* *in Eq*. (5).

#### Proof

Applying Itô’s formula ([29], Theorem 5.3.8) to the JR-NMM in Eq. (6) and using the commutativity of *M* and $\mathrm {e}^{Mt}$ we obtain

$$\begin{aligned} d \mathrm {e}^{-Mt}X(t)&=\mathrm {e}^{-Mt}\,dX(t)-M\mathrm {e}^{-Mt}X(t)\,dt \\ &=\mathrm {e}^{-Mt}\bigl(N\bigl(t,X(t)\bigr)\,dt+S(t)\,dW(t)\bigr), \end{aligned}$$

which reads in integral form

$$X(t)=\mathrm {e}^{Mt}X(0)+ \int_{0}^{t} \mathrm {e}^{M(t-s)}N\bigl(s,X(s) \bigr)\,ds+ \int_{0}^{t} \mathrm {e}^{M(t-s)}S(s)\,dW(s). $$

Since the nonlinear part *N* only depends on *Q* the equation for *Q* is given by Eq. (8). □

#### Remark 1

From the latter proof we also get a convolution-based representation for *P*, however, this formula depends on *Q*. Indeed, for $t\in[0,T]$,

$$P(t)=\vartheta'(t)Q_{0}+\kappa'(t)P_{0}+ \int_{0}^{t} \kappa'(t-s) G \bigl(s,Q(s)\bigr)\,ds+ \int_{0}^{t} \kappa'(t-s) \varSigma(s)\,dW(s). $$

#### Remark 2

System (1) has originally been deduced by using convolutions of impulse response functions with functions of the output from other subpopulations within the neural mass (see [13, 18, 37, 38]). These response functions have the same shape as the kernel *κ* in Eq. (8), which thus can be interpreted as the stochastic version of this kernel representation.

### Moment Bounds

Using representation (8) we provide bounds on the first and second moment of *Q*; analogous results can be derived for *P*. In the remainder of this section we will perform various componentwise calculations and estimations. For ease and consistency of notation we define the following: Let $x,y\in\mathbb{R}^{n}$, then $x\leq_{\odot}y$ denotes $x_{i}\leq y_{i}$ for all $1\leq i\leq n$. Furthermore, for $U,V\in\mathbb{R}^{n\times k}$ we denote the *Hadamard product* of *U* and *V* as $U\odot V$, which is defined as the elementwise product (see [39, 40]) such that each element of the $n\times k$ matrix $U\odot V$ is given as

$$(U\odot V )_{ij}=U_{ij}V_{ij} \quad \text{for } 1\leq i\leq n, 1\leq j\leq k. $$

In addition, we define $U^{2\odot}:= U\odot U$ and $U^{(1/2)\odot}$ as the elementwise root with $(U^{(1/2)\odot})_{ij}=\sqrt{U_{ij}}$.

#### Theorem 4.2

*Let* $\mu_{i}:[0,T]\rightarrow \mathbb {R}^{+}$ *for* $i\in\{3,4,5\}$ *be nonnegative functions bounded by* $\mu_{i,\max}\in\mathbb{R}^{+}$, *respectively*, *and* $C_{G}:=(Aa(\mu_{3,\max}+\nu_{\max}), Aa(\mu_{4,\max}+C_{2}\nu_{\max}),Bb(\mu _{5,\max}+C_{4}\nu_{\max}))^{T}$. *Then* $\mathbb{E}[Q(t)]$ *is bounded in each component by*

$$\vartheta(t)Q_{0}+\kappa(t)P_{0}\leq_{\odot} \mathbb {E}\bigl[Q(t)\bigr]\leq_{\odot} \vartheta (t)Q_{0}+ \kappa(t)P_{0}+\varGamma^{-2} \bigl(\mathbb{I}_{3}- \vartheta(t) \bigr) C_{G}. $$

#### Proof

We write

$$Q(t)=\underbrace{\vartheta(t)Q_{0}+\kappa(t)P_{0}}_{=:u(t)}+ \underbrace{ \int _{0}^{t} \kappa(t-s) G\bigl(s,Q(s) \bigr)\,ds}_{=:v(t)}+\underbrace{ \int_{0}^{t} \kappa (t-s)\varSigma(s)\,dW(s)}_{=:w(t)}. $$

Note that $\mathbb {E}[u(t)]=u(t)$ and that the expectation of an Itô integral is zero, i.e. $\mathbb {E}[w(t)]=0_{6}$. Recall that $\operatorname {Sigm}:\mathbb {R}\rightarrow[0,\nu_{\max}]$, thus $0_{3}\leq_{\odot} G(t,Q(t))\leq _{\odot}C_{G}$ and also $0_{3}\leq_{\odot} \mathbb {E}[G(t,Q(t))]\leq_{\odot} C_{G}$. Applying the latter bounds to $\mathbb {E}[v(t)]=\int_{0}^{t} \kappa(t-s) \mathbb {E}[G(s,Q(s))]\,ds$ and integration of *κ* yield the desired estimates. □

Obviously, the bounds provided by Theorem 4.2 also hold for the deterministic equation (3) which justifies our claim in the introduction.

#### Remark 3

The upper bound depends linearly on $\mu_{i,\max}$ and the connectivity constants $C_{i}$ whereas $u(t)$ decays exponentially fast towards 0_3_. In particular,

$$0_{3}\leq_{\odot} \lim_{t\to\infty} \mathbb {E}\bigl[Q(t) \bigr]\leq_{\odot} \varGamma ^{-2} C_{G}. $$

Similar calculations can be done for the second moments of the components of $Q(t)$. We obtain the following result.

#### Theorem 4.3

*Let the assumptions of Theorem* 4.2 *hold and assume* $\varSigma (t)$ *to be a constant matrix*, $\varSigma\in \mathbb {R}^{3\times3}$. *We define for* $x=(x_{1},x_{2},x_{3})^{T}\in \mathbb {R}^{3}$ *the function* $\mathbf{1}_{\odot }^{+}(x):=(\mathbf{1}^{+}(x_{1}),\mathbf{1}^{+}(x_{2}),\mathbf{1}^{+}(x_{3}))^{T}$, *where* $\mathbf{1}^{+}$ *denotes the indicator function of the set* $\mathbb {R}^{+}$. *Using the functions* *u*, *v* *and* *w* *from Theorem* 4.2, *a bound for the second moment of each component of* $Q(t)$ *reads*

$$\begin{aligned} &\mathbb {E}\bigl[Q^{2\odot}(t)\bigr] \\ &\quad \leq_{\odot} u^{2\odot}(t)+2u(t) \odot\mathbf {1}_{\odot}^{+}\bigl(u(t)\bigr)\odot\varGamma^{-2} \bigl(\mathbb{I}_{3}-\vartheta(t) \bigr)C_{G} \\ &\qquad {} + \biggl[\varGamma^{-2} \bigl(\mathbb{I}_{3}-\vartheta(t) \bigr)C_{G}+\frac{1}{2}\varGamma^{-3/2}\varSigma \bigl( \mathbb{I}_{3}+\kappa (t)\vartheta'(t)- \vartheta^{2}(t) \bigr)^{1/2} 1_{3} \biggr]^{2\odot}. \end{aligned}$$

*In particular*,

$$\begin{aligned} \lim_{t\to\infty} \mathbb {E}\bigl[Q^{2\odot}(t)\bigr]& \leq_{\odot} \biggl(\varGamma ^{-2}C_{G}+ \frac{1}{2}\varGamma^{-3/2}\varSigma1_{3} \biggr)^{2\odot}. \end{aligned}$$

#### Proof

From the proof of Theorem 4.2 it immediately follows that

9a $$\begin{aligned} \mathbb {E}\bigl[u^{2\odot}(t)\bigr]&=u^{2\odot}(t),\qquad \mathbb {E}\bigl[v^{2\odot}(t) \bigr]\leq\varGamma ^{-4} \bigl(\mathbb{I}_{3}-\vartheta(t) \bigr)^{2} C_{G}^{2\odot}\quad \text{and} \end{aligned}$$

9b $$\begin{aligned} \mathbb {E}\bigl[w^{2\odot}(t)\bigr]&=\frac{1}{4}\varGamma^{-3} \varSigma^{2} \bigl(\mathbb {I}_{3}+\kappa(t) \vartheta'(t)-\vartheta^{2}(t) \bigr)1_{3}. \end{aligned}$$

The last equality can be shown by applying the Itô isometry. For notational simplicity we omit the dependence on *t* in the following. By using the Cauchy–Schwarz inequality we bound

$$\mathbb {E}[v\odot w]\leq_{\odot} \bigl(\mathbb {E}\bigl[v^{2\odot}\bigr]\odot \mathbb {E}\bigl[w^{2\odot}\bigr] \bigr)^{(1/2)\odot} . $$

Applying the bounds (9a)-(9b), the desired result follows from

$$\begin{aligned} \mathbb {E}\bigl[Q^{2\odot}\bigr] =&u^{2\odot}+\mathbb {E}\bigl[v^{2\odot}\bigr]+ \mathbb {E}\bigl[w^{2\odot}\bigr]+2u\odot \mathbb {E}[v]+2\mathbb {E}[v\odot w] \\ \leq_{\odot}& u^{2\odot}+2u\odot\mathbf{1}_{\odot}^{+}(u) \odot \mathbb {E}[v] \\ &{}+ \bigl[ \bigl(\mathbb {E}\bigl[v^{2\odot}\bigr] \bigr)^{(1/2)\odot}+ \bigl(\mathbb {E}\bigl[w^{2\odot}\bigr] \bigr)^{(1/2)\odot} \bigr]^{2\odot}. \end{aligned}$$

 □

**Fig. 3:**
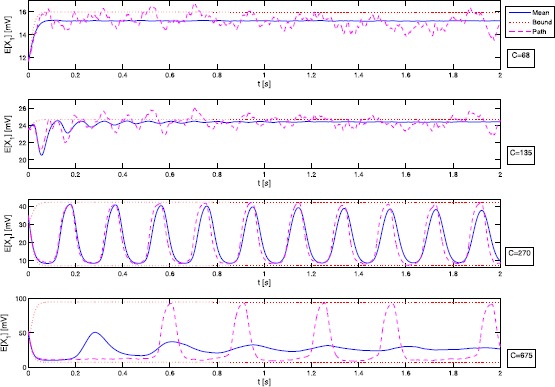
Time evolution of $\mathbb{E}[X_{1}]$

**Fig. 4:**
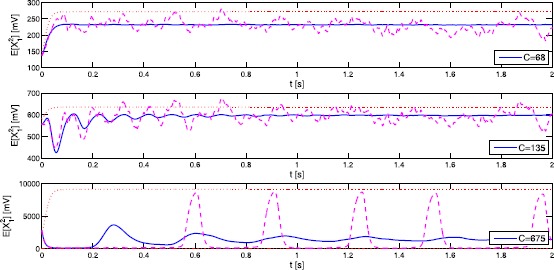
Time evolution of $\mathbb{E}[X^{2}_{1}]$

In Fig. 3 we employ Monte Carlo simulation to estimate $\mathbb{E}[X_{1}(t)]$ for varying coupling parameter *C*. The results for the second moment $\mathbb{E}[X^{2}_{1}(t)]$ are essentially the same; see Fig. 4. Similar results can be obtained for $X_{2}$ and $X_{3}$. The numerical approximations of the expectation (blue curves) stay well within the theoretical bounds (red curves), whereas single trajectories (purple curves) of course may exceed the bounds of the average. Note that, for $C=68,135$ and 675, the approximations of $\mathbb{E}[X_{1}(t)]$ rapidly converge towards fixed values for growing *t*. The same behaviour can be observed for $C=270$ on larger time scales. We will give a theoretical explanation for this phenomenon in Sect. 5 when we study the long-time behaviour of Eq. (5).

### Pathwise Bounds

Theorem 4.2 states that on average the solution of Eq. (5) stays in some bounded set. However, the theorem gives no information for single solution paths, which can in principle attain arbitrarily large values with positive probability; see Lemma A.2 in the Appendix. In this section we want to quantify the probability of such large values. The following theorem provides an upper bound on the escape probability of the components of *Q*, i.e. the probability that for $i\in\lbrace0,1,2\rbrace$ the solution $X_{i}$ is larger than a given threshold $x_{i}^{th}\in \mathbb {R}^{+}$.

#### Theorem 4.4

*Let the assumptions of Theorem* 4.3 *hold*. *For fixed* $t\in [0,T]$ *we define a Gaussian random vector* $Y(t)=(Y_{0}(t),Y_{1}(t),Y_{2}(t))$ *with*

$$\begin{aligned} \mathbb {E}\bigl[Y(t)\bigr]&=u(t)+\varGamma^{-2} \bigl(\mathbb{I}_{3}- \vartheta(t) \bigr) C_{G} \quad \textit{and} \\ \operatorname{Cov}\bigl[Y(t)\bigr]&=\frac{1}{4}\varGamma^{-3} \varSigma^{2} \bigl(\mathbb {I}_{3}+\kappa(t) \vartheta'(t)-\vartheta^{2}(t) \bigr), \end{aligned}$$

*where its components* $Y_{i}(t)$ *are independent*. *Let* $F_{Y_{i}(t)}$ *denote the cumulative distribution function of* $Y_{i}(t)$. *Then the probability that the components* $X_{i}(t)$ *for* $i\in\lbrace0,1,2\rbrace$ *exceed the given thresholds* $x^{th}_{i}\in \mathbb{R}^{+}$ *is bounded by*

$$\begin{aligned} \mathbb {P}\bigl(X_{i}(t)\ge x^{th}_{i}\bigr) \leq1-F_{Y_{i}(t)}\bigl(x^{th}_{i}\bigr). \end{aligned}$$

#### Proof

From Eq. (8), the bound on *G* and again integrating *κ*, we immediately see that each path of *Q* is bounded by the stochastic process *Y* defined by

$$Y(t)=u(t)+\varGamma^{-2} \bigl(\mathbb{I}_{3}-\vartheta(t) \bigr) C_{G}+ \int _{0}^{t} \kappa(t-s)\varSigma \,dW(s). $$

The process $Y(t)$ is Gaussian distributed with mean $u(t)+\varGamma ^{-2} (\mathbb{I}_{3}-\vartheta(t) ) C_{G}$ and covariance matrix $\frac{1}{4}\varGamma^{-3}\varSigma^{2} (\mathbb{I}_{3}+\kappa(t)\vartheta '(t)-\vartheta^{2}(t) )$, which were calculated in Theorems 4.2 and 4.3. Then, for each $t\in[0,T]$,

$$\mathbb {P}\bigl(X_{i}(t)\ge x^{th}_{i}\bigr)\leq \mathbb {P}\bigl(Y_{i}(t)\ge x^{th}_{i}\bigr)= 1-F_{Y_{i}(t)}\bigl(x^{th}_{i}\bigr). $$

 □

#### Remark 4

Theorem 4.4 can, for example, be used for calibration of the noise parameters in *Σ*. Let $\varSigma=\operatorname {diag}[\sigma_{3},\sigma_{4},\sigma_{5}]$. Suppose we want to choose $\sigma_{3}$ such that the corresponding component $X_{0}(t)$ stays below some given threshold $x_{0}^{th}$ with high probability *α*. Then a suitable choice of $\sigma_{3}$ is implicitly given by $F_{Y_{0}(t)} (x_{0}^{th} )=\alpha$.

**Fig. 5:**
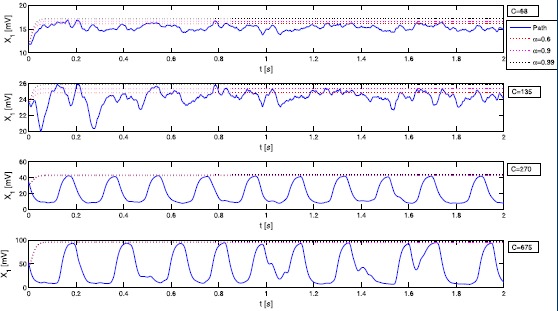
Pathwise bounds of $X_{1}$

In Fig. 5 we illustrate numerical trajectories of Eq. (5) and the corresponding bounds for varying levels of *α*, i.e. for a given time point *t*, the probability of $X_{1}(t)$ to be below the red, purple and black curve is at least 60, 90 and 99 percent, respectively.

## Long-Time Behaviour and Stationary Solutions

A further property of interest of an SDE concerns the asymptotic behaviour of solution trajectories. The classical approach in ODE theory for analysing the long-time behaviour of ODE systems is to study the stability of equilibrium solutions and limit cycles. Even in the simplest case of constant input $p(t)=p\in\mathbb{R}$, the deterministic equation (3) can possess several equilibrium solutions (both stable and unstable) as well as limit cycles with typically nontrivial basins of attraction (again we refer to the bifurcation analyses in [18, 19, 24]). Thus, the choice of the initial value can have large impact on the long-time behaviour of the solution curves of Eq. (3). From a practical point of view, this fact may be problematic, as it is not at all obvious how to estimate the initial value of the *P*-component.

In this section, we will analyse a stochastic counterpart of equilibrium solutions, more precisely invariant measures, and study the long-time asymptotics of Eq. (5). Our main tool is the theory of ergodic Markov processes, for convenience of the reader we recapitulate the basic definitions.

Let $(X(t))_{t\in[0,T]}$ be the solution process of Eq. (5). Standard stochastic analysis shows that $X(t)$ is a Markov process and the corresponding transition probability $\mathbb {P}_{t}(\mathcal {A},x)$, i.e. the probability that $X(t)$ reaches a Borel-set $\mathcal {A}\subset\mathbb{R}^{6}$ at time *t*, when it started in the point $x\in \mathbb{R}^{6}$ at time $t=0$, is given by

$$\mathbb{P}_{t}(\mathcal {A},x):=\mathbb{P} \bigl(X(t)\in \mathcal {A}\vert X(0)=x \bigr). $$

We use the definition provided in [41], Chap. 2, to characterise invariant measures. For simplicity, let ***η*** be a probability measure on $(\mathbb{R}^{6},\mathcal{B}(\mathbb{R}^{6}))$ (in general ***η*** can degenerate on some lower dimensional space). The measure ***η*** is called *invariant* if

$$\boldsymbol {\eta }(\mathcal {A})= \int_{{\mathbb {R}^{6}}}\mathbb{P}_{t}(\mathcal {A},x)\boldsymbol {\eta }(dx)\quad \forall \mathcal {A}\in \mathcal{B}\bigl(\mathbb{R}^{6}\bigr), \forall t \in[0,T]. $$

In particular, if we set the initial value $(Q_{0},P_{0})$ to be a random vector with distribution ***η***, then there exists a Markov process $X(t)$ which satisfies Eq. (5) and the distribution of $X(t)$ is ***η*** for all $t\in[0,T]$. In this sense, the concept of invariant measures can be seen as a natural extension of stationary solutions of deterministic ODEs.

We are interested in the following questions:

(i) Does Eq. (5) have an invariant measure?
(ii) Is the invariant measure unique?
(iii) Do quantities of the type $\mathbb {E}[h(X(t))]$ converge towards stationary values for a suitable class of functions $h:\mathbb {R}^{6}\to \mathbb {R}$ and any initial value $(Q_{0},P_{0})$?

We answer these questions in two steps: In the first step we will show that Eq. (5) fulfills a Lyapunov condition ensuring the existence of (possibly many) invariant measures. The questions (ii) and (iii) will be answered positively via the concept of geometric ergodicity. In classical mechanics and molecular dynamics, equations with a similar structure as Eq. (5), termed Langevin equations in the corresponding literature (see e.g. [16, 31, 36]), are well studied and we make use of relevant results concerning the long-time behaviour. In particular, we follow the presentation in [34].

As in the bifurcation analyses for Eq. (3) mentioned before, we assume that the deterministic parts of the perturbation as well as the diffusion matrix are constant, i.e.

10 $$\begin{aligned} \mu_{i}(t):=\mu_{i}\in\mathbb{R},\quad \text{for } i\in\lbrace3,4,5\rbrace\quad \text{and}\quad \varSigma(t)=\varSigma\in\mathbb {R}^{3\times3}. \end{aligned}$$

Thus, *G* does not depend on *t* and we simply write $G(Q)$.

### Existence of Invariant Measures and Geometric Ergodicity

The existence of invariant measures for Eq. (5) can be established by finding a suitable Lyapunov function. Heuristically speaking, the existence of a Lyapunov function ensures both that the solution trajectories stay in some bounded domain (except for some rare excursions) and in the case of excursions, the trajectories return to the bounded set. The following lemma shows that a perturbed version of the Hamiltonian *H* in Eq. (5) can act as a Lyapunov function (see [42]).

#### Lemma 5.1

*Assume* $a,b>0$ *and let for* $n\in\mathbb{N}$

$$\begin{aligned} V_{n}(Q,P):= \biggl(1+\frac{1}{2}\Vert P\Vert _{\mathbb{R}^{3}}^{2}+\frac{3}{2}\Vert \varGamma Q \Vert _{\mathbb{R}^{3}}^{2}+\langle P,\varGamma Q\rangle \biggr)^{n},\quad n \in\mathbb{N.} \end{aligned}$$

*Then* $V_{n}$ *is a Lyapunov function for Eq*. (5) *in the following sense*:

(i) $V_{n}\geq1$ *and* $V_{n}\rightarrow\infty$ *for* $\Vert (Q,P)^{T}\Vert _{\mathbb{R}^{6}}\rightarrow\infty$,
(ii) $\exists\alpha_{n}<0$, $\beta_{n}>0$ *such that*
  $$\begin{aligned} \mathcal{L}V_{n}\leq\alpha_{n} V_{n}+ \beta_{n}, \end{aligned}$$
  *where* $\mathcal{L}$ *denotes the generator of Eq*. (5),
  $$\mathcal{L}:=P^{T}\nabla_{Q}+ \bigl[-Q^{T} \varGamma^{2}-2P^{T}\varGamma +G(Q)^{T} \bigr] \nabla_{P}+\frac{1}{2}\sum_{i=3}^{5} \sigma_{i}^{2}\frac {\partial^{2}}{\partial X_{i}^{2}}. $$
  *Here*, $\nabla_{Q}$ *and* $\nabla_{P}$ *denote the gradient with respect to the* *Q* *and* *P* *component*, *respectively*.

#### Proof

For $a,b>0$, Property (i) is satisfied by construction and we only have to prove (ii). In a first step we set $n=1$ and analyse the action of $\mathcal{L}$ on $V_{1}$. Note that $V_{1}$ is quadratic, therefore the second derivatives in $\mathcal{L}$ result in constants. Since $P^{T}\varGamma^{2} Q=Q^{T}\varGamma^{2} P$, we obtain

11 $$\begin{aligned} \mathcal{L}V_{1} =&P^{T} \bigl(3 \varGamma^{2} Q+\varGamma P \bigr) \\ &{}+ \bigl[-Q^{T} \varGamma^{2}-2P^{T}\varGamma+G(Q)^{T} \bigr] (P+ \varGamma Q )+\frac{1}{2}\sum_{i=3}^{5} \sigma_{i}^{2} \\ =&-P^{T}\varGamma P-Q^{T}\varGamma^{3} Q + \frac{1}{2}\sum_{i=3}^{5}\sigma _{i}^{2}+G(Q)^{T} (P+\varGamma Q ). \end{aligned}$$

The first two terms in Eq. (11) are quadratic and non-positive. As $V_{1}$ is quadratic and non-negative, there always exist constants $\widetilde{\alpha}<0$ and $\widetilde{\beta}>0$ such that the first three terms in Eq. (11) can be bounded in the following way:

$$\begin{aligned} -P^{T}\varGamma P-Q^{T}\varGamma^{3} Q+ \frac{1}{2}\sum_{i=3}^{5}\sigma _{i}^{2}\leq\widetilde{\alpha}V_{1}(Q,P)+ \widetilde{\beta}. \end{aligned}$$

Furthermore, Young’s inequality implies

$$G(Q)^{T}(P+\varGamma Q)\leq\frac{\epsilon}{2} \bigl(\Vert P\Vert _{\mathbb{R}^{3}}^{2}+\Vert \varGamma Q\Vert _{\mathbb{R}^{3}}^{2} \bigr)+\frac{1}{2\epsilon}\bigl\Vert G(Q)\bigr\Vert _{\mathbb{R}^{3}}^{2}, $$

where $\epsilon>0$ can be chosen arbitrarily small. Thus, there exist $\widetilde{\alpha}<\alpha<0$ and $\beta>0$ sufficiently large such that

12 $$\begin{aligned} \mathcal{L}V_{1}(Q,P)\leq\alpha V_{1}(Q,P) + \beta. \end{aligned}$$

In a second step, we will extend this procedure to $V_{n}$ for $n\in \mathbb{ N}$. For brevity we use the notation

$$\mathcal{L}_{2}:=\frac{1}{2}\sum_{i=3}^{5} \sigma_{i}^{2}\frac{\partial ^{2}}{\partial X_{i}^{2}}\quad \text{and}\quad \mathcal{L}_{1}:=\mathcal {L}-\mathcal{L}_{2}. $$

$\mathcal{L}_{1}$ is a first-order differential operator and the action of $\mathcal{L}_{1}$ on $V_{n}$ can be bounded in the following way by using Eq. (12):

$$\mathcal{L}_{1}V_{n}=nV_{n-1} \mathcal{L}_{1}V_{1}\leq\alpha nV_{n}+n\beta V_{n-1}. $$

Applying $\mathcal{L}_{2}$ to $V_{n}$ leads to

$$\begin{aligned} \mathcal{L}_{2}V_{n}&=\frac{n}{2}\sum _{i=3}^{5}\sigma_{i}^{2} \biggl(V_{n-1}\frac{\partial^{2}V_{1}}{\partial X_{i}^{2}}+(n-1)V_{n-2} \biggl( \frac{\partial V_{1}}{\partial X_{i}} \biggr)^{2} \biggr) \\ &=\frac{n}{2}\sum_{i=3}^{5} \sigma_{i}^{2} \bigl(V_{n-1}+(n-1)V_{n-2}(X_{i}+ \varGamma_{i-2,i-2}X_{i-3})^{2} \bigr), \end{aligned}$$

where $\varGamma_{i,j}$ denotes the entry of *Γ* at the intersection of the *i*th row and *j*th column. Note that

$$\sum_{i=3}^{5}\sigma_{i}^{2}(X_{i}+ \varGamma_{i-2,i-2}X_{i-3})^{2}\leq \max\bigl\lbrace \sigma_{3}^{2},\sigma_{4}^{2}, \sigma_{5}^{2}\bigr\rbrace \Vert \varGamma Q+P\Vert _{\mathbb{R}^{3}}^{2}. $$

This implies that there exist $c,c_{n}>0$ such that

$$\begin{aligned} \frac{n(n-1)}{2}V_{n-2}\sum_{i=3}^{5} \sigma_{i}^{2}(X_{i}+\varGamma _{i-2,i-2}X_{i-3})^{2}\leq cV_{n-1}, \end{aligned}$$

and thus

$$\mathcal{L}_{2}V_{n}\leq c_{n} V_{n-1}. $$

As a consequence, there exist $\alpha_{n} <0$ (possibly close to zero) and $\beta_{n} >0$ such that

$$\begin{aligned} \mathcal{L}V_{n}=\mathcal{L}_{1}V_{n}+ \mathcal{L}_{2}V_{n}\leq\alpha nV_{n}+ (n \beta+c_{n} ) V_{n-1}\leq\alpha_{n} V_{n}+\beta_{n}, \end{aligned}$$

and Property (ii) follows. □

Lemma 5.1 has two immediate consequences. First, applying Itô’s formula on $V_{n}$ we obtain the following bounds (see [34]).

#### Corollary 5.2

*Let Assumption* (10) *hold and* $s,t\in[0,T]$ *with* $t\ge s$. *Then*

$$\mathbb{E} \bigl[V_{n}\bigl(Q(t),P(t)\bigr)\vert \mathcal{F}(s) \bigr]\leq e^{ {-\vert \alpha_{n}\vert }(t-s)}V_{n}\bigl(Q(s),P(s)\bigr)+ \frac{\beta _{n}}{\vert \alpha_{n}\vert }\bigl(1-e^{-\vert \alpha_{n}\vert (t-s)}\bigr). $$

*In particular*,

$$\mathbb{E} \bigl[V_{n}\bigl(Q(t),P(t)\bigr) \bigr]\leq e^{ {-\vert \alpha_{n}\vert }t}V_{n}\bigl(Q(0),P(0)\bigr) {+} \frac{\beta_{n}}{ {\vert \alpha_{n}\vert }}\bigl(1-e^{ {-\vert \alpha_{n}\vert }t}\bigr). $$

Second, the existence of a Lyapunov function ensures the existence of an invariant measure (see e.g. [43], Corollary 1.11).

#### Corollary 5.3

*Let Assumption* (10) *hold and let* $X(t)$ *denote the solution of Eq*. (5). *Then there exists an invariant measure* ***η*** *of* $X(t)$.

Lemma 5.1 does not give any information on the uniqueness of the invariant measure. If we further assume that the three Wiener processes $W_{i}$ act on all components of *P*, i.e. $\sigma_{i}>0$ for $i\in\lbrace3,4,5\rbrace$, we can establish the uniqueness of the invariant measure. Furthermore, the Markov process *X* fulfills the property of geometric ergodicity in the sense of [34]. We give a modification of the result in [34], Theorem 3.2, including the nonlinear function *G*.

#### Theorem 5.4

*Let* $\sigma_{i}>0$ *for all* $i\in\lbrace3,4,5\rbrace$. *The Markov process* $X(t)$ *defined by Eq*. (5) *has a unique invariant measure* ***η*** *on* $\mathbb{R}^{6}$. *Furthermore*, *let*

$$\begin{aligned} \mathcal{H}_{n}:=\bigl\lbrace h:\mathbb{R}^{6}\rightarrow \mathbb{R} \textit{ Borel-measurable with } \vert h\vert \leq V_{n}\bigr\rbrace . \end{aligned}$$

*Then for any* $n\in\mathbb{N}$ *and any initial value* $X(0)=(Q_{0},P_{0})$ *there exist positive constants* $C_{n}$, $\lambda_{n}$ *such that*

$$\begin{aligned} \biggl\vert \mathbb{E} \bigl[h\bigl(X(t)\bigr) \bigr]- \int_{\mathbb{R}^{6}}h\,d\boldsymbol {\eta }\biggr\vert \leq C_{n}V_{n} \bigl(X(0)\bigr)e^{-\lambda_{n}t}\quad \forall h \in\mathcal {H}_{n}, \forall t\geq0. \end{aligned}$$

#### Proof

The proof is the same as in [34]. The Lyapunov condition has been established in Lemma 5.1, the corresponding results for the necessary smoothness of the transition probabilities and the irreducibility of the Markov process are given in the Appendix in Lemma A.2 and A.1. Both lemmas rely on the assumption that $\sigma_{i}>0$ for $i\in\lbrace3,4,5\rbrace$. □

Theorem 5.4 has two implications for the numerical simulation of Eq. (5). First, the actual choice of the initial value is insignificant as the impact of the initial value on the distribution of $X(t)$ diminishes exponentially fast for growing *t* and an appropriate approximation of the system behaviour should be obtained with any choice of $(Q_{0},P_{0})$ provided that the system is simulated on a large enough time horizon. Second, due to the correspondence of the time averages and “space averages” of ergodic systems (see [41], Theorem 3.2.4.), one can estimate quantities of the type $\mathbb{E}[h(X(t))]$ (for *t* sufficiently large) by computing the time average of a single path of $X(t)$ on a large time horizon instead of using Monte Carlo estimation which requires one to compute a large number of paths of $X(t)$. Of course, both aspects hold only true if the numerical method reproduces the geometric ergodicity of the original system (see Sect. 6).

**Fig. 6:**
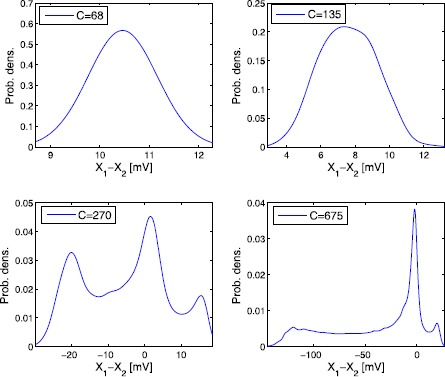
Densities of the invariant measure of *Y*

The computation of the invariant measure for nonlinear systems is highly nontrivial. One possibility would be to solve the corresponding Fokker–Planck equation, which is a six-dimensional nonlinear PDE. A standard alternative is to use stochastic simulation techniques to approximate the marginal densities of ***η***. Several possibilities have been proposed in the literature how to estimate the distribution of the solution of SDEs; see e.g. [44] for an approach based on Malliavin calculus and kernel density estimation (see [45], Chap. 2). We use the latter approach where we choose the kernel functions to be Gaussian. The numerical samples are calculated as a long-time simulation of a single path with the splitting integrator Eq. (24) proposed in Sect. 6. In Fig. 6 we compare approximations of the stationary probability density of the output signal *Y* for varying coupling parameter *C*. We observe the change from unimodal densities (for $C=68,135$) to multi-modal densities $C=270$ and to the peak-like structure $C=675$. This behaviour can be interpreted as a phenomenological stochastic bifurcation as discussed, for example, in [46] or a noise-induced transition (see [47]).

## Numerical Simulation

In order to obtain an approximation of Eq. (5) which accurately reproduces the qualitative behaviour, it is highly important to construct numerical integrators which on the one hand fulfil the properties of Eq. (5) derived in Sects. 4 and 5, and on the other hand are computationally efficient such that large ensembles of trajectories can be calculated in reasonable time.

We want to emphasise that the difficulty does not lie in the construction of a mean-square convergent integrator for Eq. (5). In fact, as the coefficient functions of Eq. (5) are globally Lipschitz continuous, any standard integrator (e.g. the Euler–Maruyama method) converges in the mean-square sense. However, it has already been shown for linear stochastic oscillators that the Euler–Maruyama method does not preserve second moment properties of that system [48] and it is expected that this negative result extends to nonlinear stochastic oscillators as well. The splitting methods fulfil the following properties:

(i) The methods preserve the moment bounds proposed in Theorems 4.2 and 4.3. Furthermore, for $\varSigma =\mathbb{O}_{3}$, the numerical method preserves the bounds of the exact solution.
(ii) The Markov process generated by the numerical method is geometrically ergodic and fulfils a Lyapunov condition under very mild step-size restrictions.

### Splitting Integrators for the JR-NMM

For convenience of the reader we provide a brief introduction to splitting integrators. Further details can be found, for example, in the classical monograph [49], Chapter II, for the deterministic case and [35, 50] for stochastic Langevin-type equations.

The main idea of splitting integrators is the following: Assume for simplicity we want to approximate a deterministic ODE system

$$\begin{aligned} \dot{y}=f(y),\qquad y(0)=y_{0}\in\mathbb{R}^{n} \end{aligned}$$

for which the function $f:\mathbb{R}^{n}\to\mathbb{R}^{n}$ can be written as

$$\begin{aligned} f(y)=\sum_{i=1}^{d}f^{[j]}(y), \quad \text{where } f^{[j]}:\mathbb {R}^{n}\to\mathbb{R}^{n} \text{ for } j\in\lbrace1,\dots, d\rbrace. \end{aligned}$$

Of course, there can be several possibilities to decompose *f*. The goal is to choose $f^{[j]}$ in such a way that the subsystems

13 $$\begin{aligned} \dot{y}=f^{[j]}(y),\quad j\in\lbrace1,\dots,d\rbrace, \end{aligned}$$

can be solved exactly. Let $\varphi^{[j]}_{t}(y_{0})$ denote the exact flow of the Subsystem (13) with initial value $y_{0}$. Then the following compositions of flows define integrators of deterministic order one (Lie–Trotter splitting) and two (Strang splitting):

$$\begin{aligned} \varPsi^{\text{LT}}_{\Delta t}(y)&= \bigl(\varphi_{\Delta t}^{[1]} \circ \cdots\circ\varphi_{\Delta t}^{[d]} \bigr) (y), \\ \varPsi^{\text{S}}_{\Delta t}(y)&= \bigl(\varphi_{\Delta t/2}^{[1]} \circ \cdots\circ\varphi_{\Delta t/2}^{[d-1]}\circ\varphi_{\Delta t}^{[d]} \circ\varphi_{\Delta t/2}^{[d-1]}\circ\cdots\circ\varphi _{\Delta t/2}^{[1]} \bigr) (y). \end{aligned}$$

This strategy can be extended to the stochastic setting (see [35] and the references therein for splitting integrators in the field of molecular dynamics, [50] for quasi-symplectic splitting integrators, [51] for variational integrators based on a splitting approach and [52] for splitting integrators in a Lie group setting). In particular, splitting integrators have been applied efficiently to Langevin equations with a similar structure as Eq. (5), see [35, 50], thus we extend this approach here to Eq. (5).

The main step in the construction of splitting integrators is to choose a suitable decomposition of the coefficient functions of Eq. (5). The right-hand side decomposes into three rather distinct parts: First, a damped, linear oscillatory part, second, a nonlinear and non-autonomous coupling part which does not depend on the *P*-component, and third, a stochastic part which does only arise in the *P*-component. Therefore,

$$\begin{aligned} \begin{pmatrix} dQ\\ dP \end{pmatrix} =\underbrace{ \begin{pmatrix} \nabla_{P} H(Q,P)\\ -\nabla_{Q}H(Q,P)- 2\varGamma P \end{pmatrix} }_{\text{damp. lin. oscill.}}\,dt+\underbrace{ \begin{pmatrix} 0_{3}\\ G(t,Q) \end{pmatrix} }_{\text{nonlin. coupl.}}\,dt+\underbrace{ \begin{pmatrix} 0_{3}\\ \varSigma(t)\,dW(t) \end{pmatrix} }_{\text{stochastic}} \end{aligned}$$

with the nonlinear term given by

$$\begin{aligned} G(t,Q)=G^{\text{I}}(t)+G^{\text{II}}(Q):= \begin{pmatrix} Aa\mu_{3}(t)\\ Aa\mu_{4}(t)\\ Bb\mu_{5}(t) \end{pmatrix} + \begin{pmatrix} Aa\operatorname {Sigm}(X_{1}-X_{2})\\ AaC_{2} \operatorname {Sigm}(C_{1} X_{0})\\ BbC_{4} \operatorname {Sigm}(C_{3} X_{0}) \end{pmatrix} . \end{aligned}$$

It makes sense to split the linear and the nonlinear drift contributions, thus providing two options to incorporate the stochastic noise. This yields two different sets of subsystems and therefore two different sets of numerical methods. In the first case, the stochastic subsystem defines a general Ornstein–Uhlenbeck process and we denote the corresponding splitting integrator as *Ornstein–Uhlenbeck integrator*. In the second case, the stochastic subsystem defines a Wiener process with drift and we denote the splitting integrator as *Wiener integrator*.

In the following let $0=t_{0}< \cdots< t_{N}=T$ with $N\in \mathbb {N}$ be an equidistant partition of $[0,T]$ with step-size Δ*t*.

### Ornstein–Uhlenbeck Integrator

The first variant is to include the stochastic contribution into the linear oscillator part, which gives rise to the two following subsystems:

14a $$\begin{aligned} \begin{pmatrix} dQ^{[1]}\\ dP^{[1]} \end{pmatrix} &= \begin{pmatrix} \nabla_{P^{[1]}} H(Q^{[1]},P^{[1]})\\ -\nabla_{Q^{[1]}}H(Q^{[1]},P^{[1]})- 2\varGamma P^{[1]} \end{pmatrix} \,dt+ \begin{pmatrix} 0_{3} \\ \varSigma(t)\,dW(t) \end{pmatrix} , \end{aligned}$$

14b $$\begin{aligned} \begin{pmatrix} dQ^{[2]}\\ dP^{[2]} \end{pmatrix} &= \begin{pmatrix} 0_{3}\\ G(t,Q^{[2]}) \end{pmatrix} \,dt. \end{aligned}$$

For both subsystems we can easily derive explicit representations of the exact solutions which can be used directly for the numerical simulation. Subsystem (14a) is a six-dimensional Ornstein–Uhlenbeck process. Let $X^{[1]}(t_{i})=(Q^{[1]}(t_{i}),P^{[1]}(t_{i}))^{T}$ denote the solution of Eq. (14a) at time point $t_{i}$ for $i\in\{0,\dots,N-1\} $, then the exact solution at time point $t_{i+1}>t_{i}$ can be represented as

15 $$\begin{aligned} X^{[1]}(t_{i+1})=e^{M\Delta t}X^{[1]}(t_{i})+ \int_{t_{i}}^{t_{i+1}} \begin{pmatrix} \kappa(t_{i+1}-s)\varSigma(s)\,dW(s)\\ \kappa'(t_{i+1}-s)\varSigma(s)\,dW(s) \end{pmatrix} , \end{aligned}$$

where $e^{M\Delta t}$ is defined in Eq. (7). $X^{[1]}$ is a Gaussian process with conditional expectation

$$\mathbb{E}\bigl[X^{[1]}(t_{i+1})\vert \mathcal{F}_{t_{i}} \bigr]=e^{M\Delta t}X^{[1]}(t_{i}) $$

and the conditional covariance matrix (see [29], Theorem 8.2.6)

$$\begin{aligned} \operatorname{Cov}(t_{i+1}) :=&\operatorname {Cov}\bigl[X^{[1]}(t_{i+1}),X^{[1]}(t_{i+1}) \vert \mathcal{F}_{t_{i}}\bigr] \\ =&\mathbb{E}\bigl[\bigl(X^{[1]}(t_{i+1})-\mathbb {E} \bigl[X^{[1]}(t_{i+1})\bigr]\bigr) \bigl(X^{[1]}(t_{i+1})- \mathbb {E}\bigl[X^{[1]}(t_{i+1})\bigr]\bigr)^{T} \vert \mathcal{F}_{t_{i}}\bigr] \\ =& \int_{t_{i}}^{t_{i+1}} e^{M(t_{i+1}-s)}\varSigma^{2}(s) \bigl(e^{M(t_{i+1}-s)}\bigr)^{T} \,ds. \end{aligned}$$

In particular, the integral term in Eq. (15) can be simulated exactly. Indeed, it is Gaussian distributed with mean zero and covariance matrix $\operatorname{Cov}(t_{i+1})$, which is for $t\ge t_{i}$ given as the unique solution of the matrix-valued ODE

16 $$\begin{aligned} \frac{d\operatorname{Cov}(t)}{dt}&=M\operatorname{Cov}(t)+\operatorname {Cov}(t)M^{T}+ \begin{pmatrix} \mathbb{O}_{3}& \mathbb{O}_{3}\\ \mathbb{O}_{3}& \varSigma^{2}(t) \end{pmatrix} , \\ \operatorname{Cov}(t_{i})&=\mathbb{O}_{6}. \end{aligned}$$

In the special case of a constant diffusion matrix $\varSigma(t)=\varSigma\in \mathbb{R}^{3\times3}$, the exact solution of Eq. (16) can be explicitly calculated for $t\ge0$ as

$$\begin{aligned} &\operatorname{Cov}(t_{i}+t) \\ &\quad = \begin{pmatrix} \frac{1}{4}\varGamma^{-3}\varSigma^{2} (\mathbb{I}_{3}+\kappa(t)\vartheta '(t)-\vartheta^{2}(t) )& \frac{1}{2}\varSigma^{2}\kappa^{2}(t)\\ \frac{1}{2}\varSigma^{2}\kappa^{2}(t) & \frac{1}{4}\varGamma^{-1}\varSigma^{2} (\mathbb{I}_{3}+\kappa(t)\vartheta'(t)-\kappa^{\prime\,2}(t) ) \end{pmatrix} . \end{aligned}$$

In general, Eq. (16) has to be solved by numerical approximation, however, it only needs to be precomputed once for the step-size Δ*t*. In either case, we obtain

17 $$\begin{aligned} X^{[1]}(t_{i+1})=e^{M\Delta t}X^{[1]}(t_{i})+ \xi_{i}(\Delta t), \end{aligned}$$

where $\xi_{i}(\Delta t)$ are iid six-dimensional Gaussian random vectors with expectation $\mathbb{E}[\xi_{i}(\Delta t)]=0_{6}$ and covariance matrix $\operatorname{Cov}(\Delta t)$.

Subsystem (14b) is a deterministic system and the solution can be obtained by integration with respect to time. As before let $X^{[2]}(t_{i})=(Q^{[2]}(t_{i}),P^{[2]}(t_{i}))^{T}$ denote the solution of Eq. (14b) at time point $t_{i}$, then the exact solution at time point $t_{i+1}>t_{i}$ is given by

18 $$\begin{aligned} Q^{[2]}(t_{i+1})&=Q^{[2]}(t_{i}), \\ P^{[2]}(t_{i+1})&=P^{[2]}(t_{i})+ \int_{t_{i}}^{t_{i+1}}G\bigl(s,Q^{[2]}(s)\bigr)\,ds \\ &=P^{[2]}(t_{i})+\Delta tG^{\text{II}} \bigl(Q^{[2]}(t_{i})\bigr)+ \int _{t_{i}}^{t_{i+1}}G^{\text{I}}(s)\,ds, \end{aligned}$$

where we assume that the last integral can be calculated exactly.

Now, let $\varphi^{\mathrm{ou},[1]}_{t}$ and $\varphi ^{\mathrm{ou},[2]}_{t}$ denote the exact flows of Eq. (14a) and (14b) given via Eq. (17) and (18), respectively. Let $x\in \mathbb{R}^{6}$, then a one-step integrator is defined by the composition of the flows

19 $$\begin{aligned} \psi^{\mathrm{ou}}_{\Delta t}(x)= \bigl( \varphi^{\mathrm{ou},[1]}_{\Delta t}\circ\varphi^{\mathrm{ou},[2]}_{\Delta t} \bigr) (x). \end{aligned}$$

### Wiener Integrator

The second possibility is to include the stochastic terms into the nonlinear contribution yielding the subsystems

20a $$\begin{aligned} \begin{pmatrix} dQ^{[1}]\\ dP^{[1]} \end{pmatrix} &= \begin{pmatrix} \nabla_{P^{[1]}} H(Q^{[1]},P^{[1]})\\ -\nabla_{Q^{[1]}}H(Q^{[1]},P^{[1]})- 2\varGamma P^{[1]} \end{pmatrix} \,dt, \end{aligned}$$

20b $$\begin{aligned} \begin{pmatrix} dQ^{[2}]\\ dP^{[2]} \end{pmatrix} &= \begin{pmatrix} 0\\ G(t,Q^{[2]}) \end{pmatrix} \,dt+ \begin{pmatrix} 0\\ \varSigma(t)\,dW(t) \end{pmatrix} . \end{aligned}$$

Subsystem (20a) is a deterministic system. Let $X^{[1]}(t_{i})=(Q^{[1]}(t_{i}),P^{[1]}(t_{i}))^{T}$ denote the solution of Eq. (20a) at time point $t_{i}$, then the exact solution at time point $t_{i+1}$ is given by

21 $$\begin{aligned} X^{[1]}(t_{i+1})=e^{M\Delta t}X^{[1]}(t_{i}). \end{aligned}$$

The solution of subsystem (20b) is—by definition—given by

22 $$\begin{aligned} Q^{[2]}(t_{i+1})={}&Q^{[2]}(t_{i}), \\ P^{[2]}(t_{i+1})={}&P^{[2]}(t_{i})+\Delta tG^{\text{II}}\bigl(Q^{[2]}(t_{i})\bigr) \\ &{}+ \int_{t_{i}}^{t_{i+1}}G^{\text{I}}(s)\,ds+ \int _{t_{i}}^{t_{i+1}}\varSigma(s)\,dW(s), \end{aligned}$$

where the last term can be simulated exactly as a three-dimensional Gaussian random vector with zero mean and covariance matrix $\int _{t_{i}}^{t_{i+1}} \varSigma^{2}(s)\,ds$. In the case of a constant diffusion matrix $\varSigma(t)=\varSigma\in\mathbb{R}^{3\times3}$, the covariance matrix is given as $\Delta t \varSigma^{2}$.

In analogy to the considerations above let $\varphi^{\mathrm{w},[1]}_{t}$ and $\varphi^{\mathrm{w},[2]}_{t}$ denote the exact flows of Eq. (20a) and (20b) given via Eq. (21) and (22), respectively. Then, for $x\in \mathbb {R}^{6}$, a one-step integrator for Eq. (5) is given by

23 $$\begin{aligned} \psi^{\mathrm{w}}_{\Delta t}(x)= \bigl( \varphi^{\mathrm{w},[1]}_{\Delta t}\circ\varphi^{\mathrm{w},[2]}_{\Delta t} \bigr) (x). \end{aligned}$$

### Order of Convergence and Strang Splitting

As the noise in Eq. (5) is additive, standard integrators such as the Euler–Maruyama method converge with mean-square order one. The same holds true for the splitting integrators constructed above.

#### Theorem 6.1

*Let* $0=t_{0}< \cdots< t_{N}=T$ *be an equidistant partition of* $[0,T]$ *with step*-*size* Δ*t*, *and let* $X^{\mathrm{ou}}(t_{i})$ *and* $X^{\mathrm{w}}(t_{i})$ *denote the numerical solutions defined by Eq*. (19) *and* (23) *at time point* $t_{i}$ *starting at initial value* $(Q_{0},P_{0})\in\mathbb{R}^{6}$. *Then the one*-*step methods defined in Eq*. (19) *and* (23) *are of mean*-*square order one*, *i*.*e*. *there exist constants* $C_{1},C_{2}>0$ *such that for sufficiently small* Δ*t* *the inequalities*

$$\begin{aligned} \mathbb{E} \bigl[\bigl\Vert X(t_{i})-X^{\mathrm{ou}}(t_{i}) \bigr\Vert _{\mathbb{R}^{6}}^{2} \bigr]^{1/2}&\leq C_{1}\Delta t, \\ \mathbb{E} \bigl[\bigl\Vert X(t_{i})-X^{\mathrm{w}}(t_{i}) \bigr\Vert _{\mathbb{R}^{6}}^{2} \bigr]^{1/2}&\leq C_{2}\Delta t, \end{aligned}$$

*hold for all time points* $t_{i}$.

#### Proof

The result can be proved in the same way as in [50], Lemma 2.1.. □

For deterministic ODE systems the convergence order of splitting methods can be increased by using composition based on fractional steps (see e.g. [49], Chapter II). We will illustrate this approach for the method based on the subsystems (20a) and (20b), the other method can be treated analogously. Using a Strang splitting we can compose the integrator

24 $$\begin{aligned} \psi^{\mathrm{w}}_{\Delta t}(x)= \bigl( \varphi^{\mathrm{w},[1]}_{\Delta t/2}\circ\varphi^{\mathrm{w},[2]}_{\Delta t} \circ\varphi^{\mathrm{w},[1]}_{\Delta t/2} \bigr) (x),\quad x\in \mathbb {R}^{6}. \end{aligned}$$

For $\varSigma={\mathbb{O}_{3}}$, Eq. (24) is a second-order method for the deterministic Eq. (2), however, the mean-square order of Eq. (24) is still one. To increase the mean-square order one has to include higher-order stochastic integrals to reproduce the interactions of the Subsystems (20a) and (20b) (see [50], Sect. 2, for details). Note that even without including the higher-order stochastic integrals the Strang splitting integrator given by Eq. (24) performs considerably better in our numerical simulations than the Lie–Trotter methods, thus we recommend to use this type of integrator. We have not yet studied the reason for this improved performance, but expect that the symmetry of the Strang splitting or the weak noise acting on the system may contribute.

**Fig. 7:**
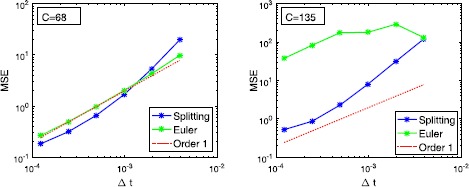
Mean-square convergence of the splitting method

We illustrate the mean-square convergence of our proposed methods in Fig. 7 and compare the Strang splitting Eq. (24) with the standard Euler–Maruyama method for the coupling parameters $C=68$ and $C=135$. As expected, both methods have mean-square order one, however, for $C=135$ the mean-square error (MSE) of the splitting method is significantly smaller than the MSE of the Euler–Maruyama method. Obviously, one might use smaller step-sizes for the Euler–Maruyama method; however, this quickly becomes highly inefficient, e.g. for the JR-NMM for multiple populations or when the Euler–Maruyama method is embedded in a continuous-time particle filter.

**Fig. 8:**
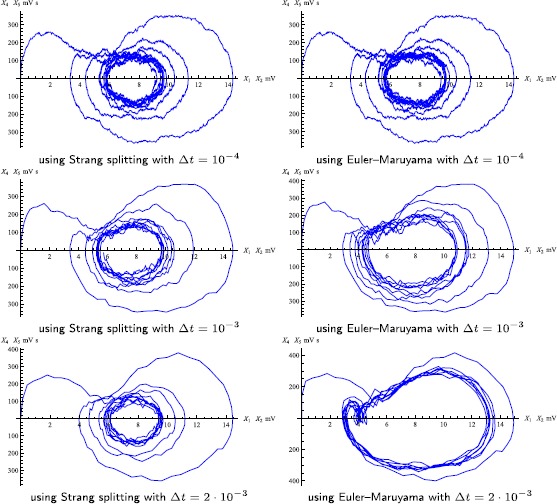
Phase portrait of one single path of *Y*

**Fig. 9:**
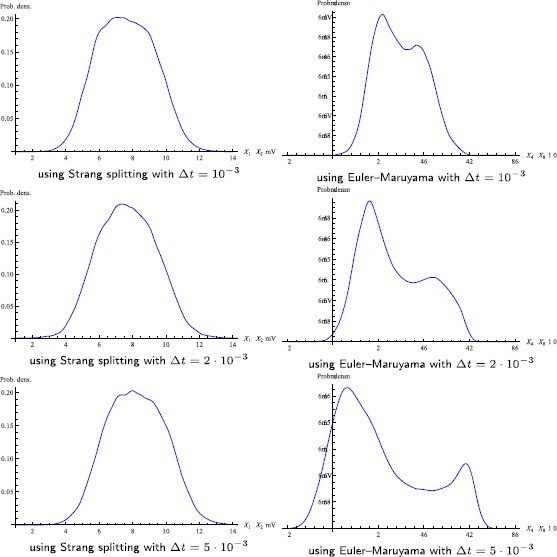
Densities of the invariant measure of *Y*

Figs. 8 and 9 also demonstrate the efficiency of the splitting scheme, as the *correct* (as not only observed from both methods with small step-sizes, but also based on our analysis of the method’s properties) results can still be produced with much larger step-sizes than those required for the Euler–Maruyama method. The other important feature of the proposed method is its reliability. Figure 8 shows several plots of the phase portrait of one single path of the output *Y*, with the splitting method and the Euler–Maruyama method and different step-sizes. It can be observed that the phase portrait obtained with the latter method changes markedly with increasing step-size. These phase portraits have been computed for the coupling parameter $C=135$, initial value $X(0)=0_{6}$, $\sigma_{3}=\sigma_{5}=1$, $\sigma_{4}=200$ and $\Delta t\in\{ 10^{-4},10^{-3},2\cdot10^{-3}\}$. Figure 9 corresponds to the upper right plot in Fig. 6, which itself can be interpreted as a computational study of a phenomenological stochastic bifurcation for varying coupling parameter *C*. It shows the densities of the invariant measure of *Y* for $C=135$, $\sigma_{3}=\sigma_{5}=10$ and $\sigma_{4}=10^{3}$ and compares the Strang splitting scheme with the Euler–Maruyama method over the time-step-sizes $\Delta t\in\{10^{-3},2\cdot10^{-3},5\cdot10^{-3}\}$. The Euler–Maruyama method with moderately small step-sizes would report a change from a unimodal to a bimodal density for the parameter $C=135$, whereas the correct value of *C* for this change to happen should be much larger.

### Moment Bounds and Geometric Ergodicity

The following two lemmas represent the properties presented in Sect. 4 for the numerical approximation schemes defined by Eq. (19) and Eq. (23). Let $X^{\text{ou}}=(Q^{\mathrm{ou}},P^{\mathrm{ou}})$ and $X^{\mathrm{w}}=(Q^{\mathrm{w}},P^{\mathrm{w}})$ denote the numerical solutions defined by Eq. (19) and Eq. (23), respectively. We start with proving analogous bounds to those in Theorem 4.2 for the expected value of $Q^{\mathrm{ou}}$ and $Q^{\mathrm{w}}$. It is well known already in the deterministic setting that the Euler scheme does not preserve such properties, see [49], Chap. 1, in the stochastic case negative results for the Euler–Maruyama method for (simple) stochastic oscillators have been observed in [48]. Note that the following two lemmas also hold when commuting the compositions in Eq. (19) and Eq. (23).

#### Lemma 6.2

*Let* $\mu_{j}:[0,T]\rightarrow \mathbb {R}^{+}$ *for* $j\in\{3,4,5\}$ *be non*-*negative functions bounded by* $\mu_{j,\max}\in\mathbb{R}^{+}$, *respectively*. *Then for* $i\in\lbrace0,\dots,N\rbrace$, $\mathbb{E}[Q^{\mathrm{w}}(t_{i})]$ (*and also* $\mathbb{E}[Q^{\mathrm{ou}}(t_{i})]$) *is bounded in each component by*

$$\begin{aligned} \vartheta(t_{i})Q_{0}+\kappa(t_{i})P_{0} \leq_{\odot}\mathbb{E}\bigl[Q^{\mathrm{w}}(t_{i})\bigr] \leq_{\odot} \vartheta(t_{i})Q_{0}+ \kappa(t_{i})P_{0}+\varGamma^{-2}C_{G}. \end{aligned}$$

#### Proof

We prove the result for the numerical method $\psi^{\mathrm{w}}$, $\psi ^{\mathrm{ou}}$ can be treated analogously. Bearing in mind the notation in Sect. 4, we obtain from Eq. (23) that

$$\begin{aligned} \mathbb {E}\bigl[X^{\mathrm{w}}(t_{i})\bigr]&=\mathrm {e}^{M\Delta t}\mathbb {E}\bigl[X^{\mathrm{w}}(t_{i-1})\bigr]+\mathrm {e}^{M\Delta t}\mathbb {E}\bigl[N \bigl(t_{i-1},X^{\mathrm{w}}(t_{i-1})\bigr)\bigr]\Delta t \\ &=\mathrm {e}^{Mt_{i}} {X(0)}+\sum_{k=1}^{i} \mathrm {e}^{Mt_{k}} \mathbb {E}\bigl[N\bigl(t_{i-k},X^{\mathrm{w}}(t_{i-k}) \bigr)\bigr]\Delta t, \end{aligned}$$

and in particular its *Q*-component reads

25 $$\begin{aligned} \mathbb {E}\bigl[Q^{\mathrm{w}}(t_{i})\bigr]= \vartheta(t_{i})Q_{0}+\kappa(t_{i})P_{0}+ \sum_{k=1}^{i}\kappa(t_{k})\mathbb {E}\bigl[G\bigl(t_{i-k},X^{\mathrm{w}}(t_{i-k})\bigr)\bigr] \Delta t. \end{aligned}$$

From the proof of Theorem 4.2 we obtain $0_{3}\leq_{\odot} \mathbb {E}[G(t_{i-k},X^{\mathrm{w}}(t_{i-k}))]\leq_{\odot} C_{G}$. Obviously, the lower bound of $\mathbb{E}[Q^{\mathrm{w}}(t_{i})]$ is fulfilled for any time step-size Δ*t*. To prove the upper bound it remains to show that

$$\sum_{k=1}^{i}\kappa(t_{k}) \Delta t\leq_{\odot}\varGamma^{-2}. $$

From the decomposition

$$\begin{aligned} \sum_{k=1}^{i}\kappa(t_{k}) \Delta t =&\bigl(\mathbb{I}_{3}-e^{-\varGamma\Delta t}\bigr)^{-1} \bigl(\mathbb{I}_{3}-e^{-\varGamma t_{i}}\bigr)\kappa(\Delta t)\Delta t- \kappa (\Delta t)\kappa(t_{i}) \\ &{}+e^{-\varGamma\Delta t}\sum _{k=1}^{i}\kappa (t_{k})\Delta t \end{aligned}$$

we derive the formula

$$\begin{aligned} \sum_{k=1}^{i}\kappa(t_{k}) \Delta t =&\bigl(\mathbb{I}_{3}-e^{-\varGamma\Delta t}\bigr)^{-2} \bigl(\mathbb{I}_{3}-e^{-\varGamma t_{i}}\bigr)\kappa(\Delta t)\Delta t \\ &{}- \bigl(\mathbb {I}_{3}-e^{-\varGamma\Delta t}\bigr)^{-1}\kappa(\Delta t)\kappa(t_{i}), \end{aligned}$$

which is in each component smaller than $\varGamma^{-2}$ for any time step-size Δ*t*. □

#### Remark 5

Another viewpoint of Eq. (23) is to apply the rectangle method (using the left boundary point of the integral) in order to approximate the convolution-based formula Eq. (8) in the form

$$\begin{aligned} Q(t+\Delta t)={}&\vartheta(\Delta t)Q(t)+\kappa(\Delta t)P(t)\\ &{}+ \int _{t}^{t+\Delta t} \kappa(t+\Delta t-s) G\bigl(s,Q(s) \bigr)\,ds \\ &{} + \int_{t}^{t+\Delta t} \kappa(t+\Delta t-s) \varSigma(s)\,dW(s). \end{aligned}$$

Moreover, Eq. (25) permits better insight into the distinction of the numerical schemes: The sum in Eq. (25) corresponds to the rectangle method in order to approximate the convolution integral $\mathbb {E}[v(t)]$ defined in the proof of Theorem 4.2, where the right boundary point is used in each approximation interval. Analogously, when commuting the composition in Eq. (23) one obtains the rectangle method evaluating the left boundary points. In the case of the Strang splitting scheme given by Eq. (24), the function *κ* is evaluated at the midpoints $(t_{k}+t_{k+1})/2$.

#### Remark 6

It can be shown analogously that the second moment $\mathbb{E}[(Q^{\mathrm{w}}(t_{i}))^{2\odot}]$ (and also $\mathbb{E}[(Q^{\mathrm{ou}}(t_{i}))^{2\odot }]$) is bounded by

$$\begin{aligned} \mathbb {E}\bigl[\bigl(Q^{\mathrm{w}}(t_{i})\bigr)^{2\odot}\bigr] \leq_{\odot}& u^{2\odot }(t_{i})+2u(t_{i})\odot \mathbf{1}_{\odot}^{+}\bigl(u(t_{i})\bigr)\odot \varGamma^{-2}C_{G} \\ &{} + \biggl(\varGamma^{-2}C_{G}+\frac{1}{2} \varGamma^{-3/2}\varSigma1_{3} \biggr)^{2\odot}. \end{aligned}$$

The last point we discuss in this article is the geometric ergodicity of the discrete Markov processes $X^{\mathrm{ou}}$ and $X^{\mathrm{w}}$ defined by Eq. (19) and (23). In analogy to Sect. 5 we assume that Assumption (10) holds. Due to the global Lipschitz continuity of the coefficients of Eq. (5), one would expect that standard numerical methods such as the Euler–Maruyama method are again geometrically ergodic for small enough step-sizes Δ*t* (see [34], Theorem 7.3). The advantage of our proposed splitting integrators is that we can directly prove a discrete analog of Lemma 5.1, i.e. a discrete Lyapunov condition for the same Lyapunov function under very mild restrictions on Δ*t*. We formulate the result for the Wiener integrator, the Ornstein–Uhlenbeck integrator can be treated analogously.

#### Lemma 6.3

*Let* $0< t_{0}\dots< t_{N}=T$ *be an equidistant partition of* $[0,T]$ *with step*-*size* $\Delta t<1/(2\Vert \varGamma \Vert _{L^{\infty}})$ *and let* $X^{\mathrm{w}}$ *denote the numerical solutions defined by Eq*. (23). *Then the functional* $V(X):=V_{1}(Q,P)$ *defined in Lemma* 5.1 *is a Lyapunov function for* $X^{\mathrm{w}}$, *i*.*e*. *there exist constants* $\alpha\in(0,1)$ *and* $\beta\ge0$ *such that*

$$\begin{aligned} \mathbb{E} \bigl[V\bigl(X^{\mathrm{w}}(t_{i+1})\bigr)\vert \mathcal{F}_{t_{i}} \bigr]&\leq\alpha V\bigl(X^{\mathrm{w}}(t_{i}) \bigr)+\beta. \end{aligned}$$

#### Proof

For the sake of simplicity we set $a=b$, which implies $e^{-\varGamma t}x=e^{-at}x$ for any $x\in\mathbb{R}^{3}$. Furthermore, we denote $Q:=Q(t_{i}),P:=P(t_{i})$ to shorten notation. The one-step approximations $Q(t_{i+1})$ and $P(t_{i+1})$ can be written as

$$\begin{aligned} \varGamma Q(t_{i+1})&=\varGamma e^{-a\Delta t}(\mathbb{I}_{3}+ \varGamma\Delta t)Q+\varGamma e^{-a\Delta t}\Delta t \bigl(P+\Delta t G(Q)+ \varSigma\xi \bigr), \\ P(t_{i+1})&=e^{-a\Delta t}(\varGamma Q+P)+e^{-a\Delta t}\Delta t G(Q)+e^{-a\Delta t}\varSigma\xi-\varGamma Q(t_{i+1}), \end{aligned}$$

where $\xi\sim\mathcal{N}(0_{3},\mathbb{I}_{3})$ is a three-dimensional Gaussian vector independent of $\mathcal{F}_{t_{i}}$. By elementary calculations and application of Young’s inequality we obtain

$$\begin{aligned} &\frac{1}{2}\bigl\Vert P(t_{i+1})+\varGamma Q(t_{i+1})\bigr\Vert ^{2}_{\mathbb{R}^{3}}\\ &\quad \leq \frac {1}{2}e^{-2a\Delta t}\Vert \varGamma Q+P\Vert ^{2}_{\mathbb{R}^{3}}+\frac{\epsilon }{2}e^{-4a\Delta t}\Delta t^{2}\Vert \varGamma Q+P\Vert ^{2}_{\mathbb{R}^{3}} \\ &\qquad {} +\frac{1}{2\epsilon}\bigl\Vert G(Q)\bigr\Vert ^{2}_{\mathbb{R}^{3}} +\frac{1}{2}\bigl\Vert e^{-a\Delta t}\bigl(\Delta tG(Q)+\varSigma\xi \bigr)\bigr\Vert ^{2}_{\mathbb{R}^{3}} \\ &\qquad {}+e^{-2a\Delta t}\langle\varGamma Q+P,\varSigma\xi\rangle_{\mathbb{R}^{3}}, \end{aligned}$$

where $\epsilon>0$ is a parameter which can be freely chosen. Thus, one can find $C_{1}>0$ sufficiently large such that

$$\begin{aligned} &\frac{1}{2}\mathbb{E} \bigl[\bigl\Vert P(t_{i+1})+\varGamma Q(t_{i+1})\bigr\Vert ^{2}_{\mathbb {R}^{3}}\vert \mathcal{F}_{t_{i}} \bigr]\\ &\quad \leq\frac{1}{2}e^{-2a\Delta t}\Vert \varGamma Q+P\Vert ^{2}_{\mathbb{R}^{3}}+\frac{1}{2\epsilon}\bigl\Vert G(Q)\bigr\Vert ^{2}_{\mathbb{R}^{3}} \\ & \qquad {}+\frac{\epsilon}{2}e^{-4a\Delta t}\Delta t^{2}\Vert \varGamma Q+P \Vert ^{2}_{\mathbb{R}^{3}}+C_{1}. \end{aligned}$$

In the same spirit we can find $C_{2}>0$ such that

$$\begin{aligned} &\mathbb{E} \bigl[\bigl\Vert \varGamma Q(t_{i+1})\bigr\Vert ^{2}_{\mathbb{R}^{3}}\vert \mathcal {F}_{t_{i}} \bigr] \\ &\quad \leq e^{-2a\Delta t}\Vert \varGamma Q\Vert ^{2}_{\mathbb {R}^{3}}+a^{2}e^{-2a\Delta t} \Delta t^{2}\Vert \varGamma Q+P\Vert ^{2}_{\mathbb{R}^{3}} \\ &\qquad {} +\tilde{\epsilon}e^{-2a\Delta t}\Delta t\Vert \varGamma Q\Vert ^{2}_{\mathbb {R}^{3}}+\frac{1}{\tilde{\epsilon}}a^{2}e^{-2a\Delta t} \Delta t\Vert \varGamma Q+p\Vert ^{2}_{\mathbb{R}^{3}}+ \frac{1}{\hat{\epsilon}} \bigl\Vert G(Q)\bigr\Vert ^{2}_{\mathbb {R}^{3}} \\ &\qquad {} +\hat{\epsilon}a^{2}e^{-4a\Delta t}\Delta t^{4} \bigl( \Vert \varGamma Q\Vert ^{2}_{\mathbb{R}^{3}}+a^{2}\Delta t^{2}\Vert \varGamma Q+P\Vert ^{2}_{\mathbb{R}^{3}} \bigr)+C_{2} \end{aligned}$$

with free parameters $\tilde{\epsilon},\hat{\epsilon}>0$. Combining the bounds above we can find a suitable $\alpha\in(0,1)$ if for any given Δ*t* there exists a choice $\tilde{\epsilon}^{*}$ such that

26a $$\begin{aligned} e^{-2a\Delta t} \biggl(1+\epsilon\Delta t^{2}+2a^{2}\Delta t^{2}+\frac {2}{\tilde{\epsilon}^{*}}a^{2}\Delta t+\hat{\epsilon} a^{4} e^{-2a \Delta t}\Delta t^{6} \biggr)&< 1, \end{aligned}$$

26b $$\begin{aligned} e^{-2a\Delta t} \bigl(1+\tilde{\epsilon}^{*}\Delta t+\hat{\epsilon }a^{2}e^{-2a\Delta t}\Delta t^{2} \bigr)&< 1. \end{aligned}$$

Note that *ϵ* and *ϵ̂* can be chosen arbitrarily small, therefore the corresponding terms can be neglected. Now let $\Delta t <1/(2a)$, then Eq. (26a) and (26b) are fulfilled for

$$\begin{aligned} \frac{2a^{2}-4a^{3}\Delta t}{2a-4a^{2}\Delta t+4a^{3}\Delta t}< \tilde{\epsilon }^{*}< \frac{2a}{1-2a\Delta t}, \end{aligned}$$

which implies the result. □

In analogy to Sect. 5, geometric ergodicity of the (discrete) Markov processes $X^{\mathrm{w}}$ and $X^{\mathrm{ou}}$ can be established by proving smoothness of the transition probabilities and irreducibility of the processes. Both properties can be proven in exactly the same way as in [34], Corollary 7.4, thus we only sketch the proof for $X^{\mathrm{w}}$:

(i) Smoothness of the transition probability densities: Due to Assumption (10) the transition probability of two (or more) consecutive steps $\psi^{\mathrm{w}}_{\Delta t}\circ\psi^{\mathrm{w}}_{\Delta t}$ of our integrator has a smooth density.
(ii) Irreducibility: As in the time-continuous case in Sect. 5 we have to establish a reachability condition, i.e. the numerical method starting at $x\in\mathbb{R}^{6}$ can reach any $y\in \mathbb{R}^{6}$ after a fixed number of steps. For our splitting method, two consecutive steps are sufficient to reach any point *y* by suitably choosing the vectors $\xi(\Delta t)$ such that
  27 $$\begin{aligned} y= \bigl(\psi^{\mathrm{w}}_{\Delta t}\circ\psi^{\mathrm{w}}_{\Delta t} \bigr) (x). \end{aligned}$$
  In fact, Eq. (27) is a six-dimensional system of equations with six degrees of freedom (three Gaussian random variables for each step $\psi^{\mathrm{w}}_{\Delta t}$) which can always be solved under Assumption 10.

To summarise, the numerical approximations $X^{\mathrm{ou}}$ and $X^{\mathrm{w}}$ are geometrically ergodic with respect to a unique invariant measure $\boldsymbol {\eta }^{\mathrm{ou}}_{\Delta t}$ and $\boldsymbol {\eta }^{\mathrm{w}}_{\Delta t}$ under mild restrictions on the time-step-size Δ*t*. Furthermore, as $X^{\mathrm{ou}}$ and $X^{\mathrm{w}}$ converge towards *X* in the mean-square sense, $\boldsymbol {\eta }^{\mathrm{ou}}_{\Delta t}$ and $\boldsymbol {\eta }^{\mathrm{w}}_{\Delta t}$ are convergent approximations of the original invariant measure ***η*** (see [53], Theorem 3.3, for details). Thus, our numerical approximations of the marginal densities in Sect. 5 (see Fig. 6) are supported by the theory.

## Summary and Conclusions

We proposed a version of the original JR-NMM incorporating random input, as a stochastic Hamiltonian system with nonlinear displacement, and discussed a range of properties based on results available in the framework of stochastic analysis, in particular properties such as moment bounds and the existence of invariant measures. The latter represent a step towards analysing the dynamical properties of a stochastic formulation of the JR-NMM. Furthermore, we presented an efficient numerical scheme based on a splitting approach which preserves the qualitative behaviour of the solution of the system. We have also discussed the advantages of applying such a scheme designed according to the obtained features of the stochastic JR-NMM for future computational studies in contrast to applying other numerical methods such as the Euler–Maruyama scheme. By a suitable introduction of noise our results can be generalised to both the extension of the JR-NMM to multiple populations [37, 54–57] and the extension to multiple areas, e.g. the 2-column model in [13] or the multi-area neural mass model in [56].

## Appendix

For completeness we give auxiliary results establishing the necessary smoothness properties of the transition probability densities as well as the irreducibility of the solution process *X* of Eq. (5). The proofs are in principle the same as in [34]. Although the Langevin equation treated there did not involve a nonlinear displacement such as the sigmoid function, the smoothness and boundedness of the sigmoid function allows us to use the same arguments. The following lemma establishes the smoothness properties of the transition probability densities by proving the hypoellipticity of the generator $\mathcal{L}$. For further details we refer to [34, 36].

### Lemma A.1

*The generator* $\mathcal{L}$, *its formal adjoint* $\mathcal{L}^{\dagger }$, $\partial/\partial t-\mathcal{L}$ *and* $\partial/\partial t-\mathcal{L}^{\dagger}$ *are hypoelliptic*.

### Proof

In order to apply Hörmander’s theorem we have to show that the Lie algebras based on the operators in question have maximal rank 6 resp. 7 (see [36] and for a more general discussion [58], Chap. 2). We show this for the generator $\mathcal{L}$, the other operators can be treated analogously. Using the notation from Sect. 4.1, we consider the following six-dimensional vector fields:

$$\begin{aligned} f_{0}(X)=MX+N(X),\qquad g_{i}= \begin{pmatrix} 0_{3}\\\varSigma_{(:,i)} \end{pmatrix} \quad \text{for } i\in\lbrace1,2,3\rbrace, \end{aligned}$$

where $\varSigma_{(:,i)}$ denotes the *i*th column of *Σ*. For six-dimensional vector fields $U,V:\mathbb{R}^{6}\to\mathbb{R}^{6}$ with Jacobians *∂U*, *∂V* we define the Lie bracket $[U,V]:=(\partial U)V-(\partial V)U$. According to [34] it suffices to show that

$$\begin{aligned} \dim \bigl(\operatorname{span} \bigl\lbrace g_{1},g_{2},g_{3},[f_{0},g_{1}],[f_{0},g_{2}],[f_{0},g_{3}] \bigr\rbrace \bigr)=6. \end{aligned}$$

As

$$\begin{aligned} (\partial f_{0})g_{i}= \begin{pmatrix} \varSigma_{(:,i)}\\ -2\varGamma\varSigma_{(:,i)} \end{pmatrix} \quad \text{and}\quad (\partial g_{i})f_{0}=0_{6}, \end{aligned}$$

the statement directly follows from Assumption (10) and $\sigma_{i}>0$ for $i\in\lbrace3,4,5\rbrace$. Note that the nonlinear term *N* does not play any role in the computation as its Jacobian is only non-zero at the derivatives corresponding to the *Q*-component, which are multiplied with the first three components of the vectors $g_{i}$. However, these three components are always zero. □

Irreducibility can be established via a control type argument. For this purpose, let $\mathbb{P}_{t}(\mathcal{A},x)$ denote the transition probabilities of the Markov process *X*.

### Lemma A.2

*The solution process* $X=(Q,P)$ *of Eq*. (5) *is irreducible*, *i*.*e*. *for arbitrary open sets* $\mathcal{A}\in\mathbb {R}^{6}$, *initial values* $x\in \mathbb {R}^{6}$ *and* $t\in[0,T]$

28 $$\begin{aligned} \mathbb{P}_{t}(\mathcal{A},x)>0. \end{aligned}$$

### Proof

It suffices to consider open neighbourhoods $\mathcal{A}=B_{\epsilon }(\mathbf{X}_{\boldsymbol{\tau}})$ of arbitrary terminal values $\mathbf{X}_{\boldsymbol{\tau}}\in\mathbb{R}^{6}$ for some time point $\tau\in[0,T]$. Now, Property (28) can be established in the following way: Let $\widehat{X}(t)$ be the solution of the controlled JR-NMM

29 $$\begin{aligned} d\widehat{X}(t)=d \begin{pmatrix} \widehat{Q}(t)\\ \widehat{P}(t) \end{pmatrix} =f_{0}(\widehat{X})\,dt+ \begin{pmatrix} {\mathbb{O}_{3}}\\ \varSigma \end{pmatrix} d\widehat{W}(t), \end{aligned}$$

where $\widehat{W}:[0,T]\to\mathbb{R}^{3}$ is a continuously differentiable function. If

30 $$\begin{aligned} \mathbb{P} \Bigl(\sup_{0\leq\tau\leq T}\bigl\Vert X( \tau)-\widehat{X}(\tau)\bigr\Vert < \epsilon \Bigr)>0, \end{aligned}$$

then Property (28) holds as long as $\widehat{X}(\tau )=\mathbf{X}_{\boldsymbol{\tau}}$. Now, let $\widehat{Q}(t)$ be a smooth curve such that

$$\begin{aligned} \begin{pmatrix} \widehat{Q}(0)\\ \frac{d\widehat{Q}}{dt}(0) \end{pmatrix} =x \quad \text{and}\quad \begin{pmatrix} \widehat{Q}(\tau)\\ \frac{d\widehat{Q}}{dt}(\tau) \end{pmatrix} =\mathbf{X}_{\boldsymbol{\tau}}. \end{aligned}$$

The control $\widehat{W}(t)$ is given via the second-order differential equation

$$\begin{aligned} \frac{d\widehat{W}}{dt}=\varSigma^{-1} \biggl(\frac{d^{2}\widehat {Q}}{dt^{2}}+2\varGamma \frac{d\widehat{Q}}{dt}+\varGamma^{2}\widehat {Q}+G(\widehat{Q}) \biggr), \end{aligned}$$

which has a unique solution as *G* is globally Lipschitz. Consequently, the point $\mathbf{X}_{\boldsymbol{\tau}}$ is reachable for solutions of the controlled System (29). Condition (30) (and therefore irreducibility) is now an immediate consequence of the Stroock–Varadhan Support Theorem (see [59]). □

## Footnotes

1 The term ’Langevin equation’ goes back to Langevin’s model of Brownian motion (1908) describing the velocity of a Brownian particle in a fluid by what is now called an Ornstein–Uhlenbeck process. However, for example, in the molecular dynamics literature the term refers to a class of models describing the interaction of heavy particles with light ones [16], whereas in some of the physics literature it may also refer to SDEs in general as in [17].
